# Differential Expression of Erythrocyte Proteins in Patients with Alcohol Use Disorder

**DOI:** 10.3390/ijms26178199

**Published:** 2025-08-23

**Authors:** İ. İpek Boşgelmez, Gülin Güvendik, Nesrin Dilbaz, Metin Esen

**Affiliations:** 1 Department of Toxicology, Faculty of Pharmacy, Erciyes University, Kayseri 38280, Türkiye; 2 Department of Toxicology, Faculty of Pharmacy, Istanbul Health & Technology University, İstanbul 34275, Türkiye; gulin.guvendik@istun.edu.tr; 3 Department of Toxicology, Faculty of Pharmacy, Ankara University, Ankara 06100, Türkiye; 4 Department of Psychology, Faculty of Humanities and Social Sciences, Üsküdar University, İstanbul 34662, Türkiye; hilmiyenesrin.dilbaz@uskudar.edu.tr; 5 Family Medicine Center, Çukurambar, Ankara 06510, Türkiye; metin_esen@yahoo.com

**Keywords:** alcohol use disorders, proteomics, erythrocyte, two-dimensional gel electrophoresis, MALDI-TOF/TOF, protein-protein interaction network, hubs

## Abstract

Alcohol Use Disorder (AUD) poses global health challenges, and causes hematological alterations such as macrocytosis and oxidative stress. Disruption of protein structures by alcohol and/or its metabolites may exacerbate AUDs; proteomics can elucidate the underlying biological mechanisms. This study examined the proteins differentially expressed in the cytosol and membrane fractions of erythrocytes obtained from 30 male patients with AUD, comparing them to samples from 15 age- and BMI-matched social drinkers (SDs) and 15 non-drinkers (control). The analysis aimed to identify the molecular differences related to alcohol consumption. The AUD patient subgrouping was based on mean corpuscular volume (MCV), with 16 individuals classified as having a normal MCV and 14 having a high MCV. Proteins were separated via two-dimensional(2D)-gel electrophoresis, digested with trypsin, and identified via Matrix-Assisted Laser Desorption/Ionization Time-of-Flight (TOF) mass spectrometry (MALDI-TOF/TOF). Additionally, levels of malondialdehyde and 4-hydroxyalkenals (MDA + HAE), reduced glutathione (GSH), oxidized glutathione (GSSG), serum carbohydrate-deficient transferrin (%CDT), disialotransferrin (%DST), and sialic acid (SA) were analyzed. The results showed increased MDA + HAE and decreased total thiols in AUD patients, with GSSG elevated and the GSH/GSSG ratio reduced in the AUD MCV-high subgroup. Serum %CDT, %DST, and SA were significantly higher in AUD. Compared to the control profiles, the AUD group exhibited differential protein expression. Few proteins, such as bisphosphoglycerate mutase, were downregulated in AUD versus control and SD, as well as in the MCV-high AUD subgroup. Conversely, endoplasmin and gelsolin were upregulated in AUD relative to control. Cytoskeletal proteins, including spectrin-alpha chain, actin cytoplasmic 2, were overexpressed in the AUD group and MCV-high AUD subgroup. Several proteins, such as 14-3-3 isoforms, alpha-synuclein, translation initiation factors, heat shock proteins, and others, were upregulated in the MCV-high AUD subgroup. Under-expressed proteins in this subgroup include band 3 anion transport protein, bisphosphoglycerate mutase, tropomyosin alpha-3 chain, uroporphyrinogen decarboxylase, and WD repeat-containing protein 1. Our findings highlight the specific changes in protein expression associated with oxidative stress, cytoskeletal alterations, and metabolic dysregulation, specifically in AUD patients with an elevated MCV. Understanding these mechanisms is crucial for developing targeted interventions and identifying biomarkers of alcohol-induced cellular damage. The complex interplay between oxidative stress, membrane composition, and cellular function illustrates how chronic alcohol exposure affects cellular physiology.

## 1. Introduction

The global burden of alcohol use disorder (AUD) is substantial, surpassing many other risk factors [1]. Alcohol-related harms pose significant public health challenges, leading to a plethora of medical, social, and economic issues worldwide. While mostly acute effects involve injuries, violence, and fatalities at high doses, chronic excessive alcohol intake is linked to various conditions, including AUD, increased cancer risk, and teratogenic effects [2]. Alcohol consumption has been associated with over 200 health conditions, such as infectious diseases, cancers, mental and behavioral disorders, neurological disorders, cardiovascular diseases, gastrointestinal disorders, and injuries [3]. Historically, the hypothesis has been that light to moderate nutritional intake could confer health benefits [4]; however, alcohol consumption is linked to considerable health risks, which sustains the ongoing debate [5,6,7]. To address these issues, the 2022–2030 Global Alcohol Action Plan aims to enhance global strategies for mitigating alcohol-related morbidity and mortality, as well as social impacts [8].

Several biomarkers, including carbohydrate-deficient transferrin (CDT), sialic acid (SA), and mean corpuscular volume (MCV), may provide relatively objective information regarding AUD [9]. Some studies have also evaluated the combined use of certain parameters [10,11,12]. On the other hand, the potential use of proteomics, which is “the systematic and large-scale analysis of the proteome, the set of proteins produced by a given cell or organism under a defined set of conditions” has been relatively new [13,14]. Studies conducted in some biological samples of AUD patients [15,16] and animal models [17,18] have revealed some promising findings. Differences in protein expression profiles related to some diseases may serve as various options, such as biomarkers applicable in diagnosis, prognosis, or treatment efficacy [19,20]. In this field, various methods are available, and two-dimensional gel electrophoresis (2-DE) followed by Matrix-Assisted Laser Desorption/Ionization (MALDI) Time-of-Flight (TOF) mass spectrometric analysis continues to offer valuable insights. After the purification or separation of proteins based on their specific isoelectric points (pI) through first-dimension isoelectric focusing (IEF), SDS-PAGE further separates them according to their molecular weight (MW).

Chronic alcohol consumption impairs erythrocyte aggregation and deformability, impacting microcirculatory function and blood rheology through various mechanisms, including disruption of membrane lipids and proteins, oxidative stress, and toxic metabolite accumulation, which compromise erythrocyte integrity and cellular function. Notably, some adverse effects may be partially reversible upon cessation of alcohol intake [21]. It is well established that the significance of erythrocytes extends beyond their essential function of transporting oxygen and carbon dioxide. Their potential, particularly as promising targets for the diagnosis of various diseases and as frameworks for developing innovative therapies, has been increasingly acknowledged [22,23,24]. Nemkov et al. (2018) [22] underscore the significance of erythrocytes in maintaining systemic metabolic homeostasis, emphasizing their interactions with various cell types, notably immune cells. They point out the therapeutic potential of targeting these interactions to develop innovative therapies for conditions including hypoxemia, inflammation, neurodegenerative disorders, aging, and cancer [22]. Similarly, Anastasiadi et al. (2024) propose that erythrocytes might function as an organ actively participating in maintaining physiological homeostasis by acting as redox and ion buffers, monitoring vascular tone, and signaling to the immune system [24]. In this vein, the extensive metabolic network within erythrocytes has been underscored, along with glycolysis, the cytosolic metabolism of tricarboxylic acids, purine, and arginine being particularly noteworthy. Furthermore, erythrocytes possess various receptors and transporters for hormones, neurotransmitters, and drugs, acting as “sinks” or transporters for a wide range of molecules. In this view, it has been highlighted that multi-omics approaches can be utilized to develop a comprehensive erythrocyte map, revealing the potential of erythrocytes as systemic sensors of disease and modulators of physiological processes, thereby advancing erythrocyte-based biomedical applications [24]. Pretini et al. (2019) also highlighted the critical aspects of erythrocyte functions and interactions within the context of cellular and molecular biology [23]. As outlined, erythrocytes participate in complex interactions with various cellular components, including endothelial cells, platelets, leukocytes, and macrophages, primarily through direct receptor-mediated contact. Furthermore, they engage indirectly via plasma-derived ligands and proteins, underscoring the elaborate nature of their involvement in physiological processes. Additionally, their mechanical behavior under physiological flow underscores their role beyond mere contact, illustrating the complexity of erythrocyte interactions. Their inherent ability to deform and aggregate significantly impacts blood viscosity, which in turn influences blood flow across various shear rates. This dynamic property is essential for maintaining normal circulatory function and demonstrates how alterations in these characteristics can affect overall cardiovascular health. In pathological conditions such as sickle cell disease, type 2 diabetes, and malaria, erythrocytes often become more adhesive and less deformable, leading to microvascular obstructions that impair oxygen and nutrient delivery to organs. Erythrocyte microparticles may contribute to pro-inflammatory cytokine secretion, oxidative stress, and endothelial apoptosis, which can lead to vaso-occlusive crises [23].

Considering all these details and the progression of scientific research into a new phase characterized by integrated approaches, most notably exemplified by the introduction of the “exposome” concept in toxicology, including the “alcohol exposome,” the field is rapidly transforming into extensive integration. The alcohol exposome encompasses factors such as comorbidities, smoking, diet, occupational and environmental exposures, infectious agents, and age. It underscores the importance of intercellular communication among organs or systems, which influences alcohol-related injuries and metabolite profiles. Future research may prioritize inter-organ signaling via exosomes, which transfer cargos including proteins through endocytosis or receptor-mediated entry, thereby delivering molecules capable of activating specific kinases in distant tissues [25].

This study investigates the effects of alcohol and its metabolites on erythrocyte proteins using 2-DE and MALDI-TOF-TOF MS/MS, comparing profiles among AUD patients, social drinkers, and nondrinkers. Given the variability in MCV levels among AUD patients, a comparative analysis of erythrocyte protein expression was conducted between those with high versus normal MCV levels, including social drinkers and controls. Additionally, functional annotation and protein–protein interactions of erythrocyte cytosolic and membrane fractions were examined in relation to differentially expressed proteins. The findings are expected to enhance the understanding of how chronic alcohol intake influences erythrocyte protein expression, emphasizing the molecular functions most affected by alcohol and contributing to knowledge of the hematological alterations associated with alcohol consumption.

## 2. Results

The study groups (Table 1) comprised male participants matched for age and BMI. When assessing ethanol consumption, only the AUD subgroups exhibited statistically significant differences compared to controls and social drinkers; other groups did not show such differences. Although participants in the MCV-high subgroup demonstrated higher ethanol intake (232.0 ± 95.07 g ethanol per day) compared to the MCV normal subgroup (203.0 ± 98.86 g ethanol per day), the MCV-normal and MCV-high subgroups did not differ significantly in ethanol intake. While the MCV-high subgroup exhibited the highest mean values in MCV and AST/ALT, along with reduced RDW and folic acid levels, these differences were not statistically significant across groups. Notably, the vitamin B_12_ levels in the MCV-high subgroup were higher than those in controls, social drinkers, and the AUD-MCV-normal subgroup, with the latter group also showing elevated B_12_ compared to controls. GGT levels, which were highest in the MCV-high subgroup, followed a similar trend to that of vitamin B_12_.

**Figure 1:**
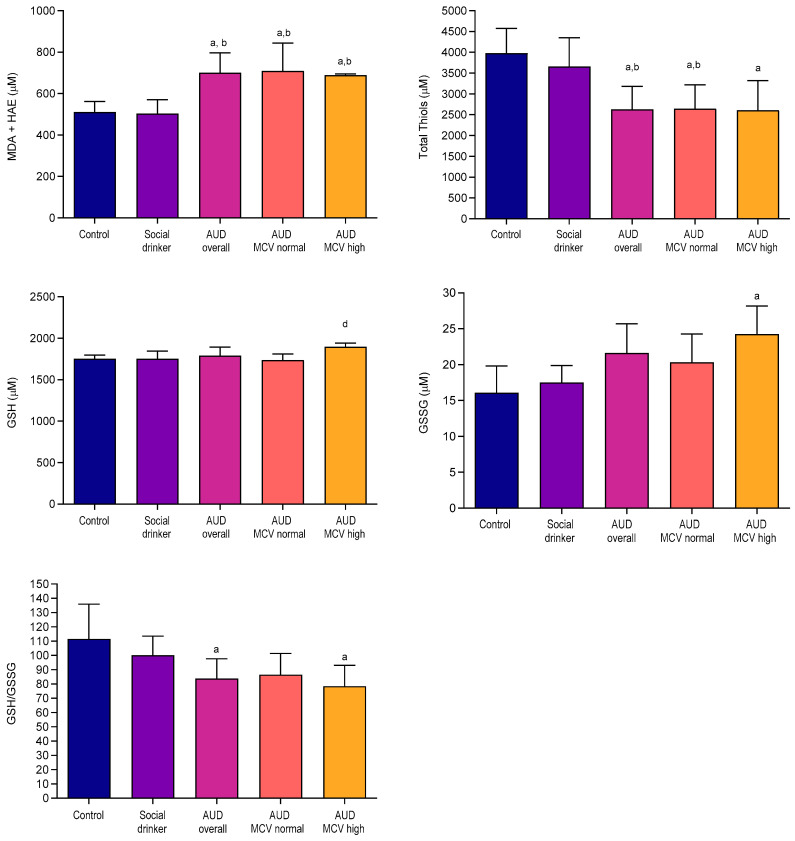
Oxidative stress and total thiols, glutathione (total, reduced, oxidized) status in the erythrocyte samples of the study groups. ^a^ *p* < 0.05 as compared to control; ^b^ *p* < 0.05 as compared to SD; ^d^ *p* < 0.05 as compared to the AUD-MCV-normal subgroup.

As illustrated in Figure 1, levels of lipid peroxidation markers, malondialdehyde (MDA), and hydroxyalkenal (HAE) in erythrocyte hemolysates from patients with AUD were significantly elevated compared to the controls and social drinkers (*p* < 0.05). Furthermore, within the AUD group, MDA + HAE levels were higher in individuals with and without macrocytosis relative to the control and social drinker groups. A similar increase was observed in GSSG levels among AUD patients. Conversely, total thiol levels and the GSH/GSSG ratio were markedly reduced in erythrocytes from AUD patients. The concurrent rise in GSH and GSSG in the high MCV AUD subgroup may represent an adaptive response.

**Figure 2:**
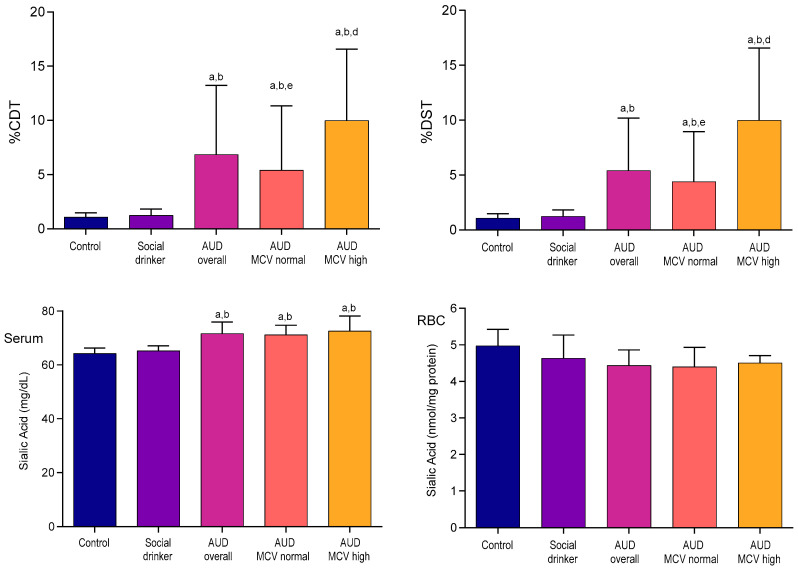
Carbohydrate-deficient transferrin (CDT), disialotransferrin (DST) in serum, and sialic acid (serum and erythrocyte SA) contents in study groups. ^a^ *p* < 0.05 as compared to control; ^b^ *p* < 0.05 as compared to SD; ^d^ *p* < 0.05 as compared to AUD-MCV-normal subgroup; ^e^ *p* < 0.05 as compared to AUD-MCV-high subgroup.

Figure 2 shows that in the AUD groups, %CDT and disialotransferrin, one of the isoforms particularly emphasized in recent studies, were increased compared to the control and social drinker groups (*p* < 0.05). Most notably, the subgroup of MCV-high presented the highest levels. As expected, the serum SA levels were notably elevated in individuals with AUD compared to the controls and social drinkers, with no significant differences among patient subgroups. Conversely, erythrocyte SA levels appeared lower in patients, but one-way ANOVA indicated that these differences were not statistically significant (*p* > 0.05).

**Figure 3:**
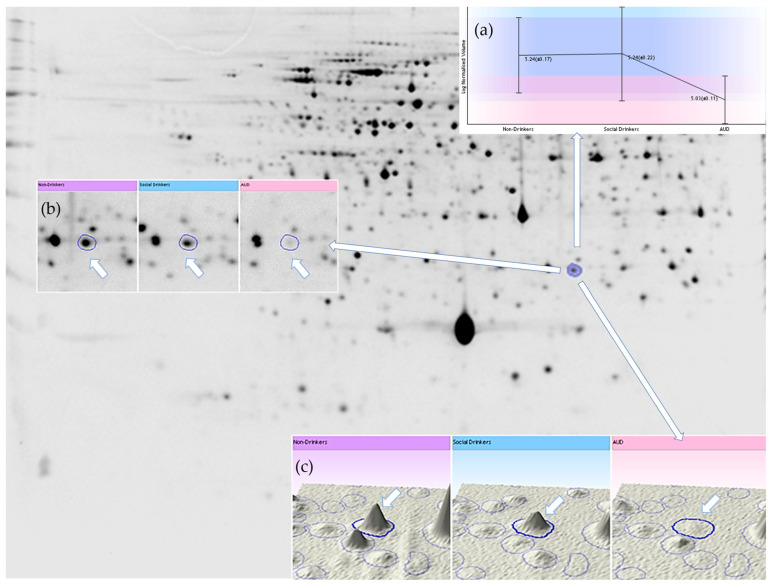
Representative gel image of the cytosolic fraction of the erythrocyte sample (IPG strip: 24 cm, pH 4–7, 100 μg protein/gel, software: Progenesis SameSpots, v 4.0). (**a**) Log-normalized volume of a selected spot (bisphosphoglycerate mutase), (**b**) 2D image of spot, (**c**) 3D image of spot.

**Figure 4:**
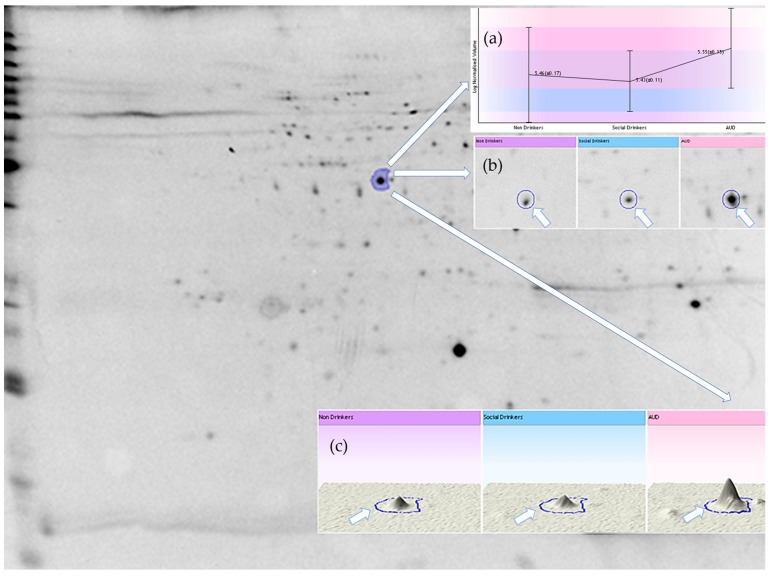
Representative gel image of the membrane fraction of the erythrocyte sample (IPG strip: 24 cm, pH 4–7, 100 μg protein/gel, software: Progenesis SameSpots, v 4.0). (**a**) Log-normalized volume of a selected spot (actin), (**b**) 2D image of spot, (**c**) 3D image of spot.

**Figure 5:**
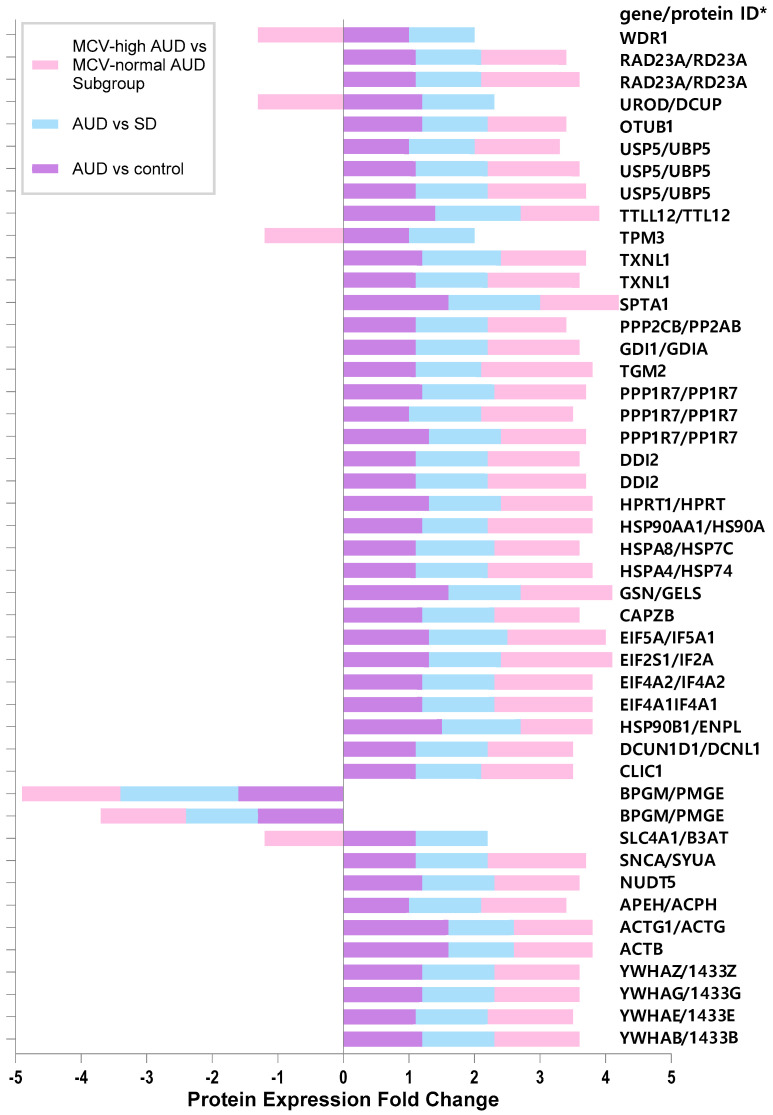
A brief representation of fold changes in the cytosolic and membrane proteins with differential expression in the study groups. Protein accession information, gene names, and additional details are available in Table 2. * If the gene and protein IDs are different, the names are listed with the gene ID first, followed by the protein ID.

Protein expression analysis (Figure 3 and Figure 4) demonstrated that, relative to the control subjects, certain protein spots, especially within the MCV-high subgroup gels of the AUD group, exhibited differential expression. As presented in Figure 5 and Table 2:

- Expressions of endoplasmin and gelsolin were upregulated in AUD compared to the controls.
- Cytoskeletal proteins such as spectrin alpha chain, and actin cytoplasmic 1 and 2 were overexpressed in the AUD group and the MCV-high AUD subgroup.
- The MCV-high AUD subgroup compared to the MCV-normal AUD subgroup had significantly higher expression of 14-3-3 protein beta/alpha, 14-3-3 protein gamma, 14-3-3 protein zeta/delta, F-actin-capping protein subunit beta, hypoxanthine-guanine phosphoribosyl-transferase, protein phosphatase 1 regulatory subunit 7, thioredoxin-like protein 1, UV excision repair protein RAD23 homolog A, eukaryotic translation initiation factor 2 subunit 1 (IF2A), protein glutamine gamma-glutamyltransferase 2 (TGM2), heat shock protein HSP 90-alpha (HSP90A), heat shock 70 kDa protein 4 (HSP74), alpha-synuclein (SYUA), eukaryotic translation initiation factor 5A-1 (IF5A1), eukaryotic initiation factor 4A-II (IF4A2), eukaryotic initiation factor 4A-I (IF4A1), protein DDI1 homolog 2 (DDI2), and ubiquitin carboxyl-terminal hydrolase 5 (UBP5).
- Conversely, bisphosphoglycerate mutase (PMGE) was consistently downregulated across all AUD groups, specifically in the MCV-high AUD subgroup.
- Additionally, four proteins, namely WD repeat-containing protein 1 (WDR1), uroporphyrinogen decarboxylase (DCUP), tropomyosin alpha-3 chain (TPM3), and band 3 anion transport protein (B3AT), exhibited differential expression only in the MCV subgroup comparison, with downregulation observed in the MCV-high subgroup relative to MCV-normal AUD patients.

**Table 2:** Cytosolic and membrane proteins with differential expression in AUD patients.

Proteins	Spot No. ^a^	Accession ID	Protein MW (kDa)/pI (Theoretical)		Protein MW (kDa)/pI (Observed)		Peptide Count	Protein Score	Protein Score C.I. %	Total Ion Score	Total Ion C.I. %	Change (Fold) ^b^		
												AUD vs. Control	AUD vs. SD	MCV-High AUD vs. MCV-Normal AUD
14-3-3 protein beta/alpha OS = *Homo sapiens* GN = YWHAB PE = 1 SV = 3	C590	1433B	28.065	4.8	28	4.67	16	555	100	432	100	↑ 1.2	↑ 1.1	↑ 1.3 *
14-3-3 protein epsilon OS = *Homo sapiens* GN = YWHAE PE = 1 SV = 1	C559	1433E	29.155	4.6	29	4.53	19	619	100	474	100	↔ 1.1	↔ 1.1	↑ 1.3 *
14-3-3 protein gamma OS = *Homo sapiens* GN = YWHAG PE = 1 SV = 2	C576	1433G	28.285	4.8	28	4.68	11	416	100	351	100	↑ 1.2	↔ 1.1	↑ 1.3 *
14-3-3 protein zeta/delta OS = *Homo sapiens* GN = YWHAZ PE = 1 SV = 1	C589	1433Z	27.728	4.7	28	4.62	17	775	100	645	100	↑ 1.2	↔↑ 1.1	↑ 1.3 *
Actin, cytoplasmic 1 OS = *Homo sapiens* GN = ACTB PE = 1 SV = 1	C472	ACTB	41.710	5.3	38	5.51	7	403	100	377	100	↑ 1.6 *	↔ 1.0	↔ **↑ ** **1.2**
Actin, cytoplasmic 2 OS = *Homo sapiens* GN = ACTG1 PE = 1 SV = 1	C807	ACTG	41.766	5.3	44	5.25	16	654	100	546	100	↑ 1.6 *	↔ 1.0	**↑ 1.2**
Acylamino-acid-releasing enzyme OS = *Homo sapiens* GN = APEH PE = 1 SV = 4	C110	ACPH	81.173	5.3	82	5.35	24	1040	100	887	100	↔ 1.0	↔ 1.1	↑ 1.3 *
ADP-sugar pyrophosphatase OS = *Homo sapiens* GN = NUDT5 PE = 1 SV = 1	C529	NUDT5	24.312	4.9	33	4.73	8	538	100	488	100	↑ 1.2	↔ 1.1	↑ 1.3 *
Alpha-synuclein OS = *Homo sapiens* GN = SNCA PE = 1 SV = 1	C706	SYUA	14.451	4.7	17	4.64	3	235	100	219	100	↔ 1.1	↔↓ 1.1	↑ 1.5 *
Band 3 anion transport protein OS = *Homo sapiens* GN = SLC4A1 PE = 1 SV = 3	M126	B3AT	101.727	5.1	45	4.44	14	839	100	794	100	↔↓ 1.1	↔↓ 1.1	↓ 1.2 *
Bisphosphoglycerate mutase OS = *Homo sapiens* GN = BPGM PE = 1 SV = 2	C582	PMGE	29.987	6.1	28	6.18	11	480	100	405	100	↓ 1.3 *	↔↓ 1.1	↓ 1.3 *
Bisphosphoglycerate mutase OS = *Homo sapiens* GN = BPGM PE = 1 SV = 2	C588	PMGE	29.987	6.1	28	5.98	7	158	100	122	100	↓ 1.6 *	↓ 1.8 *	↓ 1.5 *
Chloride intracellular channel protein 1 OS = *Homo sapiens* GN = CLIC1 PE = 1 SV = 4	C558	CLIC1	26.906	5.1	30	5.14	14	680	100	568	100	↔ 1.1	↔ 1.0	↑ 1.4 *
DCN1-like protein 1 OS = *Homo sapiens* GN = DCUN1D1 PE= 1 SV =1	C600	DCNL1	30.105	5.2	27	5.11	7	145	100	116	100	↔ 1.1	↔ 1.1	↑ 1.3 *
Endoplasmin OS = *Homo sapiens* GN = HSP90B1 PE = 1 SV = 1	C46	ENPL	92.411	4.8	100	4.77	14	181	100	145	100	↑ 1.5 *	↑ 1.2	↔ 1.1
Eukaryotic initiation factor 4A-I OS = *Homo sapiens* GN = EIF4A1 PE = 1 SV = 1	C373	IF4A1	46.125	5.3	47	5.27	23	846	100	677	100	↑ 1.2	↔↑ 1.1	↑ 1.5 *
Eukaryotic initiation factor 4A-II OS = *Homo sapiens* GN = EIF4A2 PE = 1 SV = 2	C376	IF4A2	46.373	5.3	47	5.36	16	608	100	513	100	↑ 1.2	↔ 1.1	↑ 1.5 *
Eukaryotic translation initiation factor 2 subunit 1 OS = *Homo sapiens* GN = EIF2S1 PE =1 SV=3	C475	IF2A	36.089	5.0	37	5.13	16	387	100	285	100	↑ 1.3	↔ 1.1	↑ 1.7 *
Eukaryotic translation initiation factor 5A-1 OS = *Homo sapiens* GN = EIF5A PE = 1 SV = 2	C716	IF5A1	16.821	5.1	16	5.09	8	580	100	528	100	↑ 1.3	↔ 1.2	↑ 1.5 *
F-actin-capping protein subunit beta OS = *Homo sapiens* GN = CAPZB PE = 1 SV = 4	C555	CAPZB	31.331	5.4	30	5.67	13	365	100	281	100	↑ 1.2	↔ 1.1	↑ 1.3 *
Gelsolin OS = *Homo sapiens* GN = GSN PE = 1 SV = 1	C74	GELS	85.644	5.9	92.0	5.8	21	554	100	443	100	**↑ 1.6**	↔ 1.1	↔↑ 1.4
Heat shock 70 kDa protein 4 OS = *Homo sapiens* GN = HSPA4 PE = 1 SV = 4	C31	HSP74	94.271	5.1	107	5.13	17	452	100	397	100	↔ 1.1	↔ 1.1	↑ 1.6 *
Heat shock cognate 71 kDa protein OS = *Homo sapiens* GN = HSPA8 PE = 1 SV = 1	C815	HSP7C	70.854	5.4	69	5.38	26	1200	100	1015	100	↔ 1.1	↔↑ 1.2	↑ 1.3 *
Heat shock protein HSP 90-alpha OS = *Homo sapiens* GN = HSP90AA1 PE= 1 SV= 5	C83	HS90A	84.607	4.9	89	4.94	22	844	100	750	100	↔↑ 1.2	↔ 1.0	↑ 1.6 *
Hypoxanthine-guanine phosphoribosyltransferase OS = *Homo sapiens* GN = HPRT1 PE = 1 SV=2	C642	HPRT	24.564	6.2	23	4.77	9	238	100	182	100	↑ 1.3 *	↔↑ 1.1	↑ 1.4 *
Protein DDI1 homolog 2 OS = *Homo sapiens* GN = DDI2 PE = 1 SV = 1	C333	DDI2	44.495	5.0	50	4.93	15	813	100	715	100	↑ 1.1	↔ 1.1	↑ 1.5*
Protein DDI1 homolog 2 OS = *Homo sapiens* GN = DDI2 PE = 1 SV = 1	C344	DDI2	44.495	5.0	50	5.05	18	928	100	795	100	↔ 1.1	↔ 1.1	↑ 1.4 *
Protein phosphatase 1 regulatory subunit 7 OS = *Homo sapiens* GN = PPP1R7 PE = 1 SV = 1	C377	PP1R7	41.539	4.8	47	4.88	20	500	100	346	100	↑ 1.3*	↔ 1.1	**↑ 1.3**
Protein phosphatase 1 regulatory subunit 7 OS = *Homo sapiens* GN = PPP1R7 PE = 1 SV = 1	C379	PP1R7	41.539	4.8	47	4.81	19	491	100	349	100	↔ 1.0	↔↓ 1.1	↑ 1.4 *
Protein phosphatase 1 regulatory subunit 7 OS = *Homo sapiens* GN = PPP1R7 PE = 1 SV = 1	C380	PP1R7	41.539	4.8	47	4.84	18	325	100	197	100	↑ 1.2	↔↓ 1.1	↑ 1.4 *
Protein-glutamine gamma-glutamyltransferase 2 OS = *Homo sapiens* GN = TGM2 PE = 1 SV = 2	C97	TGM2	77.280	5.1	87	5.11	26	940	100	761	100	↔ 1.1	↔ 1.0	↑ 1.7 *
Rab GDP dissociation inhibitor alpha OS = *Homo sapiens* GN = GDI1 PE = 1 SV = 2	C224	GDIA	50.550	5.0	64	5.0	16	220	100	139	100	↔↑ 1.1	↔ 1.1	**↑ 1.4**
Serine/threonine-protein phosphatase 2A catalytic subunit beta isoform OS = *Homo sapiens* GN = PPP2CB PE	C488	PP2AB	35.552	5.2	36	5.24	11	227	100	160	100	↔ 1.1	↔ 1.1	↑ 1.2 *
Spectrin alpha chain, erythrocyte OS = *Homo sapiens* GN = SPTA1 PE = 1 SV = 5	M112	SPTA1	280.014	5.0	50	5.17	21	104	100	86	100	↑ 1.6 *	↑ 1.4	**↑ 1.2**
Thioredoxin-like protein 1 OS = *Homo sapiens* GN = TXNL1 PE = 1 SV = 3	C504	TXNL1	32.231	4.8	36	4.89	11	653	100	581	100	↑ 1.2	↑ 1.2	↑ 1.3 *
Thioredoxin-like protein 1 OS = *Homo sapiens* GN = TXNL1 PE = 1 SV = 3	C506	TXNL1	32.231	4.8	36	4.97	12	708	100	627	100	↔ 1.1	↔ 1.1	↑ 1.4 *
Tropomyosin alpha-3 chain OS = *Homo sapiens* GN = TPM3 PE = 1 SV = 1	M174	TPM3	32.799	4.7	31	4.6	11	410	100	361	100	↔ 1.0	↔ 1.0	↓ 1.2 *
Tubulin–tyrosine ligase-like protein 12 OS = *Homo sapiens* GN = TTLL12 PE = 1 SV = 2	C519	TTL12	74.356	5.3	80	5.44	24	846	100	686	100	↑ 1.4	↑ 1.3	↑ 1.2 *
Ubiquitin carboxyl-terminal hydrolase 5 OS = *Homo sapiens* GN = USP5 PE = 1 SV = 2	C52	UBP5	95.725	4.9	96	4.92	31	1030	100	816	100	↔ 1.1	↔ 1.1	↑ 1.4 *
Ubiquitin carboxyl-terminal hydrolase 5 OS = *Homo sapiens* GN = USP5 PE = 1 SV = 2	C53	UBP5	95.725	4.9	96	4.95	34	1090	100	835	100	↔ 1.1	↔ 1.1	**↑ 1.5**
Ubiquitin carboxyl-terminal hydrolase 5 OS = *Homo sapiens* GN = USP5 PE = 1 SV = 2	C814	UBP5	95.725	4.9	96	4.9	27	714	100	550	100	↔ 1.0	↔ 1.0	↑ 1.3 *
Ubiquitin thioesterase OTUB1 OS = *Homo sapiens* GN = OTUB1 PE = 1 SV = 2	C519	OTUB1	31.264	4.9	34	4.74	15	773	100	653	100	↑ 1.2	↔ 1.0	↑ 1.2 *
Uroporphyrinogen decarboxylase OS = *Homo sapiens* GN = UROD PE = 1 SV = 2	C466	DCUP	40.761	5.8	38	5.88	8	288	100	252	100	↔↓ 1.2	↔ 1.1	**↓ 1.3**
UV excision repair protein RAD23 homolog A OS = *Homo sapiens* GN = RAD23A PE = 1 SV = 1	C283	RD23A	39.585	4.6	56	4.46	16	513	100	395	100	↔ 1.1	↔ 1.0	↑ 1.5 *
UV excision repair protein RAD23 homolog A OS = *Homo sapiens* GN = RAD23A PE = 1 SV = 1	C291	RD23A	39.585	4.6	56	4.49	15	448	100	341	100	↔ 1.1	↔ 1.0	↑ 1.3 *
WD repeat-containing protein 1 OS = *Homo sapiens* GN = WDR1 PE = 1 SV = 4	C166	WDR1	66.152	6.2	70	6.53	16	276	100	191	100	↔ 1.0	↔ 1.0	↓ 1.3 *

Automated image analysis and protein expression difference assessments were conducted using SameSpots software (Nonlinear Dynamics SameSpots v 4.0, Newcastle-Upon-Tyne, UK), ^a^ C: cytosolic fraction, M: membrane fraction, ^b^ spot volume changes ↑: increase (red), ↓: decrease (blue), ↔: no change, ↔↑: partial increase, ↔↓: partial decrease, * *p* < 0.05 MCV high vs. MCV normal (within AUD), OS: organism name, GN: gene name of the UniProtKB entry, SV: SequenceVersion, MW: molecular weight, pI: isoelectric point.

**Figure 6:**
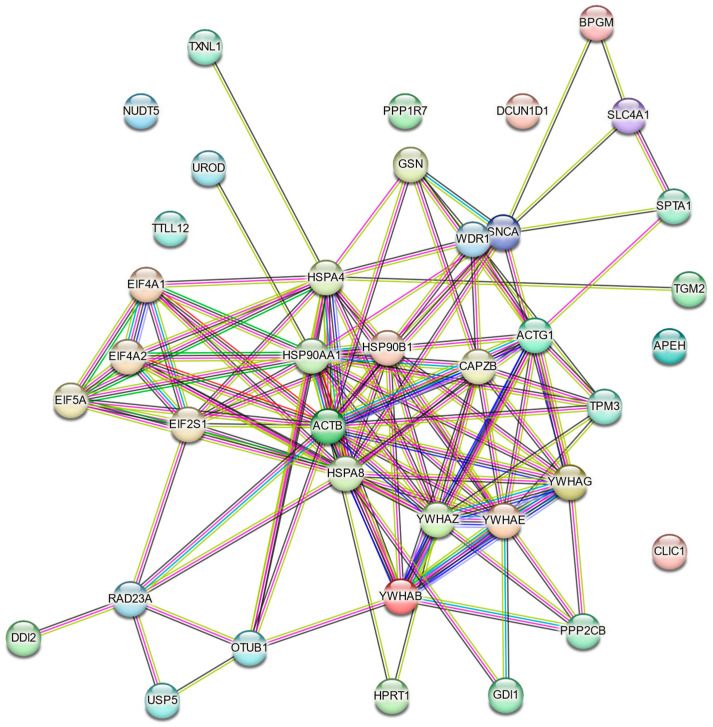
Protein–protein interaction network (retrieved from STRING [29]).

**Figure 7:**
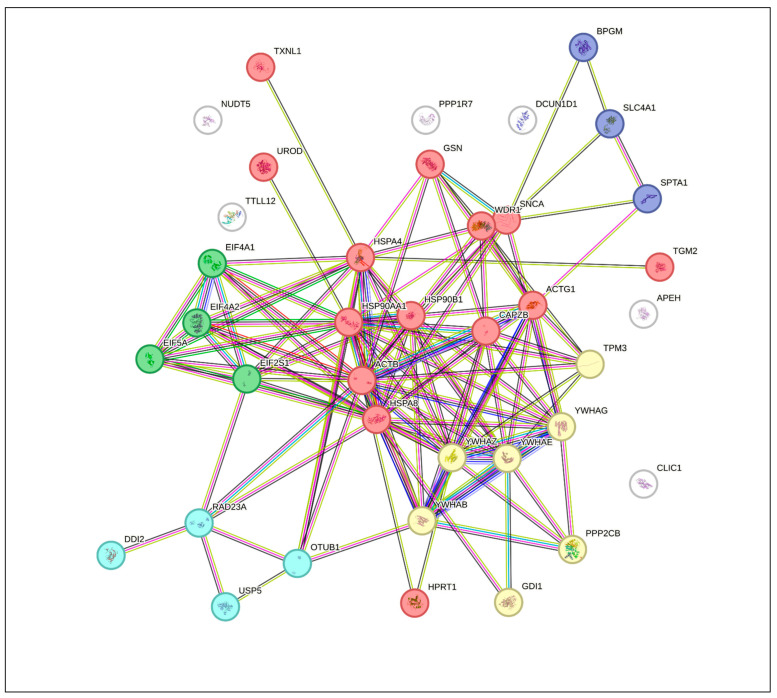

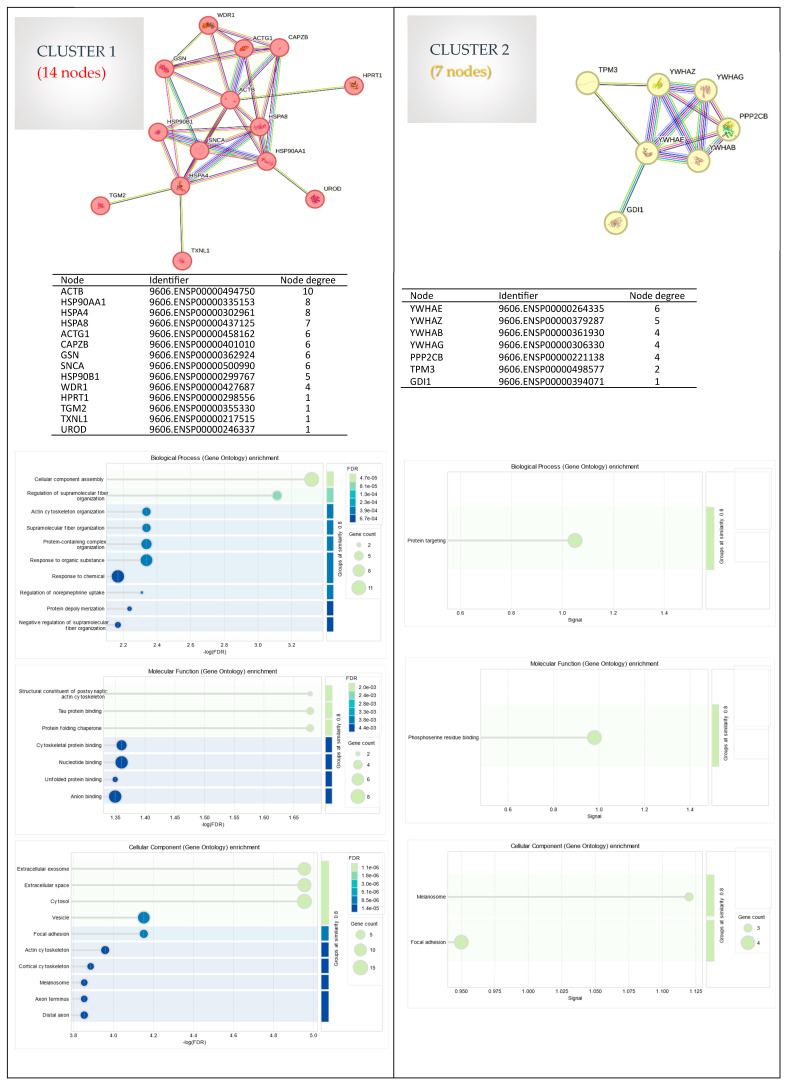

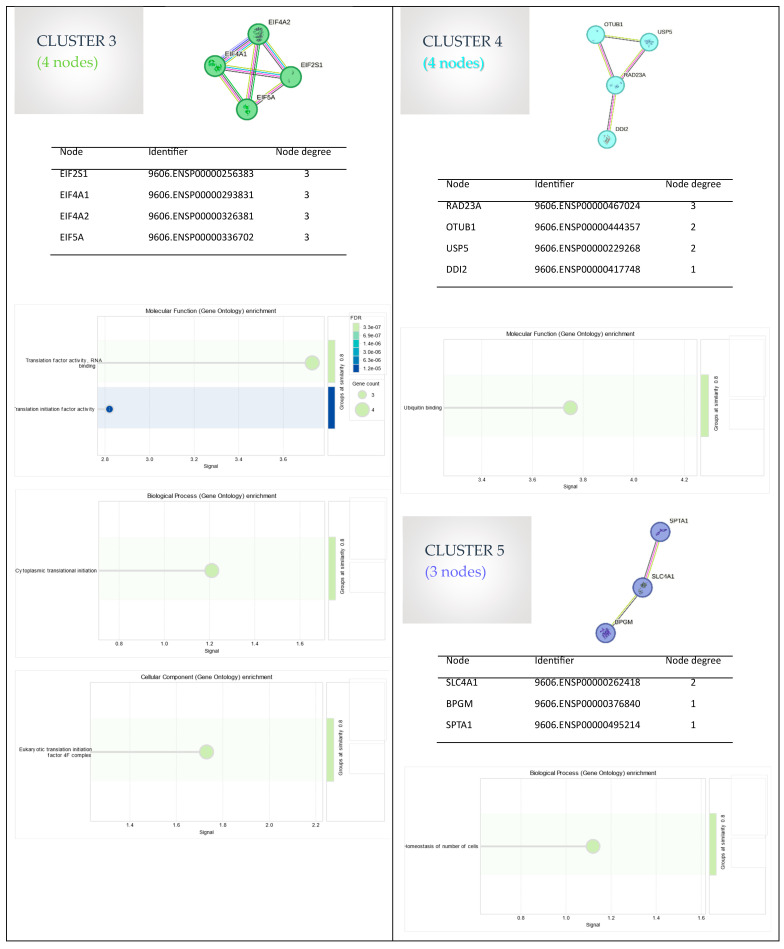
k-means clustering generated using STRING (5 clusters).

A sub-network was retrieved from the STRING database, highlighting protein interaction enrichment (Figure 6). Using STRING, we also conducted k-means clustering, identifying five clusters of interest (Figure 7). We found that a functional annotation chart obtained using DAVID Bioinformatics Resources: DAVID 2021 [26,27] revealed 125 chart records, of which 38 were statistically significant. The terms included acetylation, actin cytoskeleton, protein binding, protein sequestering activity, PI3K-Akt signaling, actin capping, and stress response. The functional annotation clustering of differentially expressed erythrocyte proteins resulted in nine clusters, of which groups 1 to 5 were significant in the Bonferroni test (*p* < 0.05). These clusters contained terms such as focal adhesion, protein sequestering activity, phosphoserine residue binding, protein targeting, ubiquitin protein ligase binding, stress response, actin cytoskeleton, and blood microparticle (Supplementary Materials: Tables S1 and S2). Using another online tool, ShinyGO [28], we also evaluated the functional enrichment as GO “Biological Process” (Table 3), “Cellular Component” (Table 4), and “Molecular Function” (Table 5).

**Figure 8:**
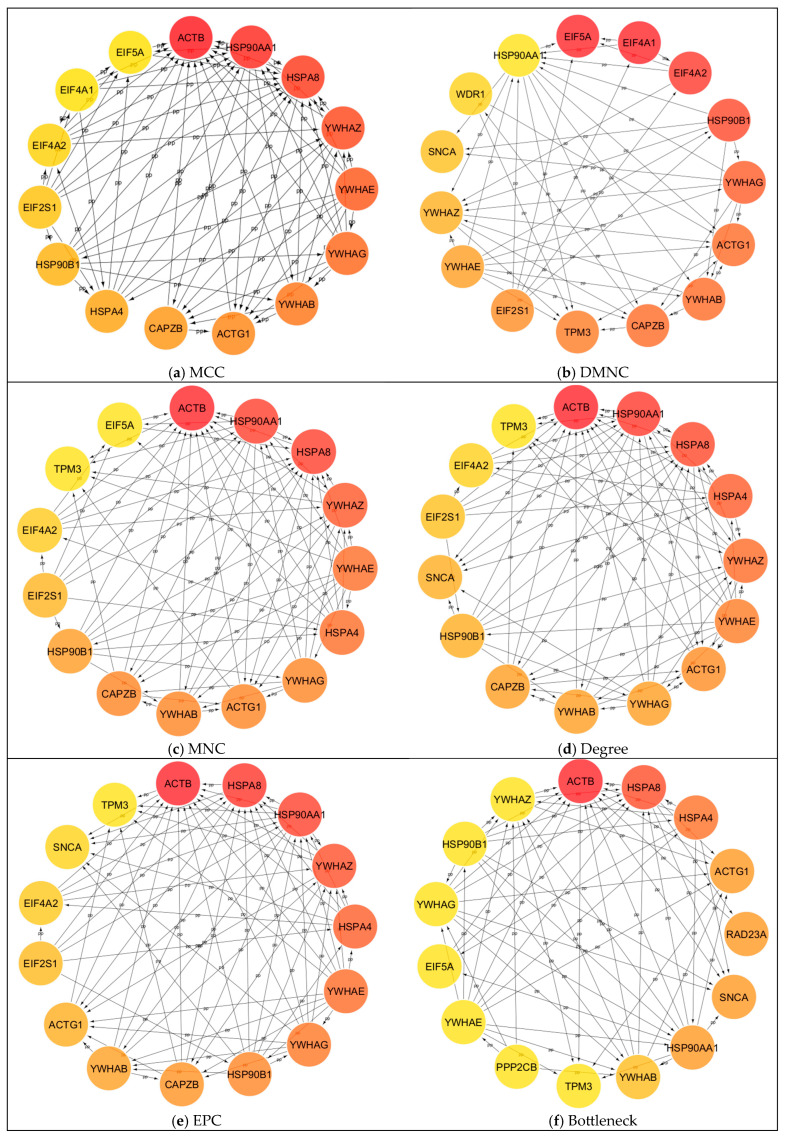

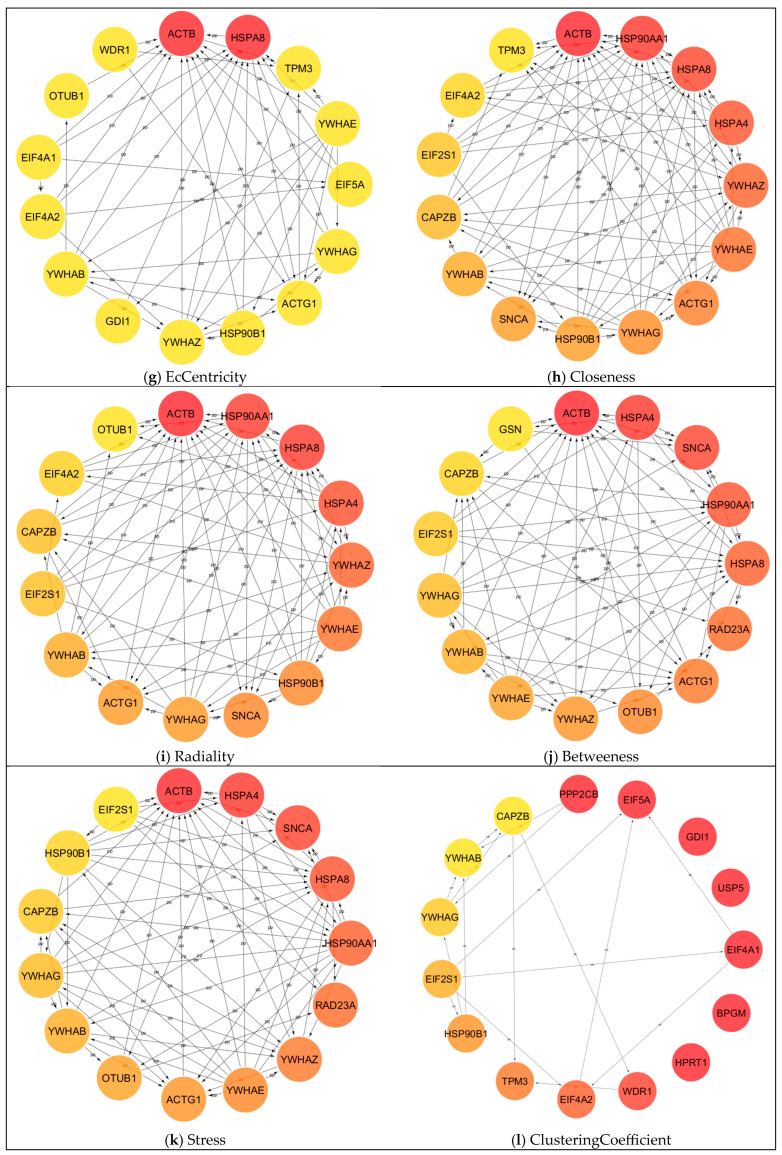
Protein–protein interaction network, hub proteins, found using CytoScape plugin cytoHubba 30.

**Figure 9:**
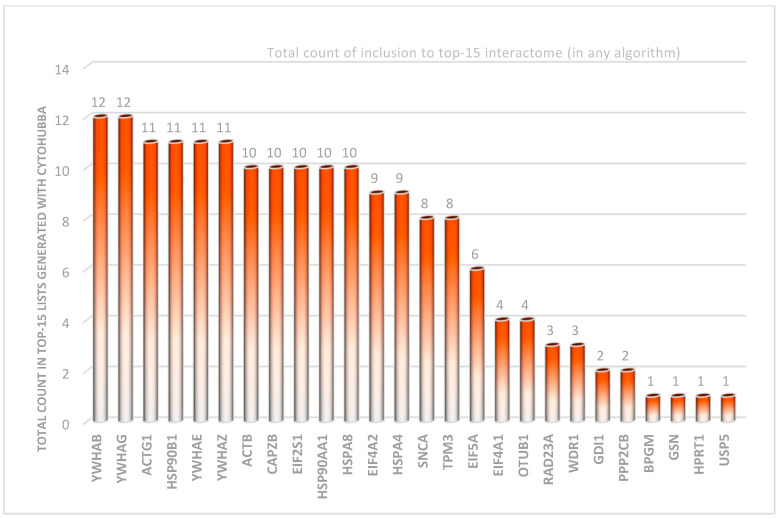
Total counts of hubs in the top-15 lists generated using cytoHubba algorithms. The numbers present the count of each hub in the algorithms. The most frequently included ones are YWHAB and YWHAG (12 times each), while the least frequently included ones are BPGM, GSN, HPRT1, and USP5.

**Figure 10:**
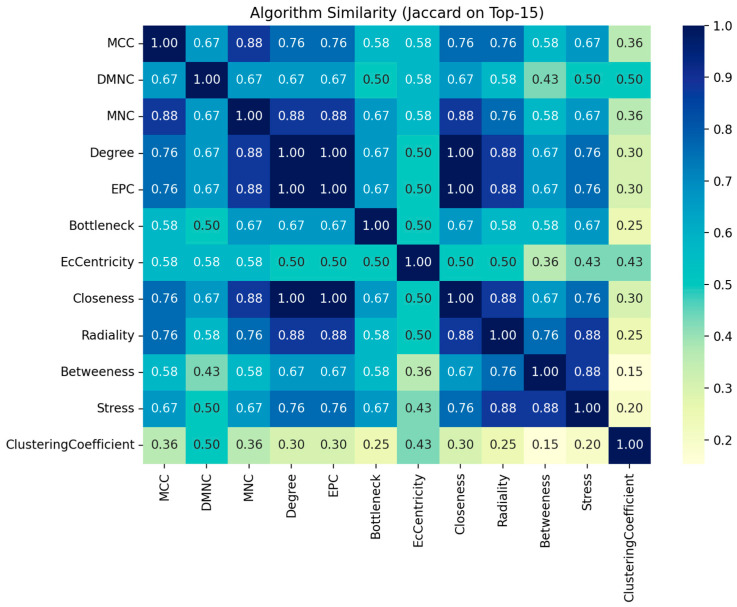
Algorithm similarity generated via Julius AI [31].

In our effort to explore the interaction between the proteins of interest and identify hub proteins within the interactome, we utilized the CytoHubba plugin in CytoScape [30]. To assess node importance within a network, this application incorporates twelve node ranking algorithms, categorized into two primary types: global-based and local-based methods. Local ranking approaches evaluate a node’s significance by analyzing its direct connections, whereas global methods consider the node’s importance within the context of the entire network. As demonstrated in Figure 8, all available ranking algorithms were applied to determine the top 15 proteins in our study. While the majority of methods yielded similar top-ranked proteins, some approaches, namely DMNC, EcCentricity, and ClusteringCoefficient, produced at least partially different sets of top proteins compared to the others. Figure 9 provides a comprehensive overview of the frequency of each protein identified across twelve distinct algorithms. The most consistently detected proteins are YWHAB and YWHAG, each appearing in all twelve analyses. Other significant hubs include ACTG1, HSP90B1, YWHAE, YWHAZ, ACTB, CAPZB, EIF2S1, HSP90AA1, HSPA8, EIF4A2, and HSPA4. In contrast, certain proteins, such as chloride intracellular channel protein 1, DCN1-like protein 1, protein DDI1 homolog 2, and protein phosphatase 1 regulatory subunit 7, were not represented in the rankings. Additionally, hubs like BPGM, GSN, HPRT1, and USP5 were each ranked only once. The similarity of algorithms generated by Julius AI [31] in Figure 10 depicts that the degree, EPC, and closeness metrics yielded the most comparable rankings. Although the primary list of items remained stable, algorithms showed differences in the ranking of hubs, as illustrated in Figure 8d,e,h).

## 3. Discussion

Erythrocytes, commonly known as red blood cells, are often perceived as simple mammalian cells; however, they demonstrate a significant degree of complexity. Some researchers propose classifying erythrocytes as an organ [22,24] considering their abundance, crucial role in systemic metabolic homeostasis, and participation in intricate metabolic processes that extend beyond oxygen and carbon dioxide transport [22]. As the primary oxygen carriers, erythrocytes are vulnerable to oxidative stress, with alcohol exposure inducing lipid peroxidation and thiol modifications, as shown in Figure 1. Mature erythrocytes are highly specialized cells that minimize energy consumption and utilize only a limited number of metabolic pathways to generate energy, which is crucial for their primary functions. However, this specialization renders erythrocytes particularly susceptible to various disorders. Metabolic processes can be influenced by the production of reactive oxygen species, and oxidative stress can impair the function of enzymes that are essential for metabolic pathways [32]. AUD is known to affect antioxidant defense and promote oxidative stress-induced damage. In a recent study, assessment of blood and urine redox status (total antioxidant capacity [TAC], total oxidative status [TOS], and oxidative stress index [OSI]) in controls, alcohol overdose, and dependency revealed significantly elevated TAC levels in both alcohol-dependent and alcohol-poisoned individuals compared to controls. TOS levels were found to be higher in both groups versus controls, with the highest in alcohol dependency. Blood TAC correlated strongly with blood alcohol content; ROC analysis showed it effectively distinguished between alcohol poisoning and dependency with high sensitivity and specificity [33]. D’Alessandro et al. [34] found a significant correlation between oxidative stress markers and AUD biomarker ethyl glucuronide levels in blood samples from a donor cohort. Specifically, erythrocytes from donors with elevated ethyl glucuronide exhibited increased glutathione synthesis and turnover into oxoproline, a downstream metabolite in mature erythrocytes due to the absence of oxoprolinase. The authors propose that markers of alcohol consumption are linked to heightened oxidative stress and reduced energy metabolism, without significantly affecting hemolytic parameters in stored erythrocytes from healthy volunteers. Despite elevated oxidative stress markers and increased levels of pentose phosphate pathway metabolites and ascorbate and its oxidized form, subjects with higher ethyl glucuronide levels were observed, indicating decreased glutathione recycling. While some studies suggest the beneficial effects of moderate alcohol on the erythrocyte deformability [35,36], in other studies, decreased membrane fluidity of erythrocytes was suggested to be related to the elevated plasma and erythrocyte nitric oxide levels [37] or peroxidative damage to membrane lipids and oxidation of membrane protein thiols [38] in chronic alcohol exposure. Another biomarker of AUD, the SA in serum samples from AUD patients were notably elevated compared to social drinkers and controls as also shown in previous studies [39,40]; however, the MCV-based subgrouping appears to have no effect on serum SA levels (Table 1). Of note, while erythrocyte SA levels appeared lower in patients, one-way ANOVA did not reveal statistically significant differences (*p* > 0.05).

The study further reveals that the AUD groups, particularly the MCV-high subgroup, show increased vitamin B_12_ levels alongside decreased folic acid levels (Table 1). It is well known that macrocytosis, characterized by MCV exceeding the normal range, affects about 90% of individuals with excessive alcohol intake, often even before the onset of anemia. Maruyama et al. (2001) [41] have reported that alcohol abuse and folic acid deficiency may contribute secondarily to macrocytosis, evidenced by higher MCV and RDW in patients with alcoholic liver disease. They found MCV to be correlated positively with alcohol intake and negatively with serum folic acid levels, while abstinence was observed to lead to significant reductions in MCV and RDW, with increases in serum folic acid [41]. Folate deficiency is rare in countries with routine food supplementation but persists among malnourished individuals with high alcohol intake and limited diets. Macrocytosis may also result from liver damage, which alters erythrocyte membrane lipid composition. Alcohol-induced macrocytosis can occur despite adequate folate and vitamin B_12_ levels and in the absence of liver disease. Although the precise mechanism remains uncertain, it may involve acetaldehyde adduct formation in erythrocyte membranes. Macrocytosis resolves within two to four months after stopping alcohol intake. Another point to consider is that iron deficiency can suppress macrocytosis since it often causes microcytosis [42]. The crosstalk between erythrocytes and other cell types, including immune cells, has also garnered interest. Niemelä et al. (2022) investigated heavy alcohol consumption’s impact on blood cell composition, identifying alterations in leukocyte and erythrocyte parameters associated with neutrophil activation, hemolysis, inflammatory mediators, and immune responses to ethanol metabolites [43]. Their results underscored that baseline MCV correlated with hemolysis markers such as LDH and bilirubin, suggesting erythrocyte susceptibility to hemolysis following episodes of heavy drinking, despite normal haptoglobin levels. Additionally, MCV was linked to IL-6, IL-8, and serum ferritin, indicating inflammation.

Proteomics might be of use to delineate the mechanisms underlying alcohol-related damage in erythrocytes and/or adaptive responses. In this regard, the functional annotation chart and clusters also provide a useful perspective (Table 3, Table 4 and Table 5 and Figure 5). Despite extensive research efforts, the exact mechanisms by which erythrocyte proteins interact with and influence vital cellular processes remain to be elucidated [44]. Even with state-of-the-art tools, detecting very low-abundance proteins is quite challenging in the presence of high-abundance proteins such as hemoglobin (cytoplasm), band-3 (membrane), and spectrin (cytoskeleton) [44,45]. In this study, we employed 2-DE and MALDI-TOF-TOF MS/MS to analyze the protein expression profiles in erythrocytes from individuals with AUD, social drinkers, and controls. Since elevated MCV is common in individuals with AUD and tends to increase with abstinence, we also compared erythrocyte protein profiles between participants with elevated and those with normal MCV, as well as social drinkers and controls. Our analysis identified significant differences in the protein expression levels within the AUD group. For example, bisphosphoglycerate mutase expression was markedly reduced in the AUD group relative to both the control and SD groups, with the reduction being most prominent in the MCV-high AUD subgroup. In an experimental study in rats, chronic alcohol ingestion was found to reduce 2,3-bisphosphoglycerate (2,3-BPG) levels and increase hemoglobin’s affinity for oxygen [46]. Bisphosphoglycerate mutase influences hemoglobin’s oxygen affinity by modulating the levels of 2,3-BPG, which is present in erythrocytes at a relatively high concentration and biosynthesized in the Luebering–Rapoport pathway mainly by bisphosphoglycerate mutase. Besides its role in oxygen affinity, 2,3-BPG may serve as a radical scavenger, capable of neutralizing hydroxyl and peroxyl radicals, chelating iron, and preventing iron oxidation within hemoglobin [47]. We observed that the expression of some other proteins, including gelsolin, was upregulated in AUD compared with the control. Gelsolin is a calcium-regulated, actin-modulating protein that prevents monomer exchange. Cytoskeletal proteins spectrin alpha chain, actin cytoplasmic 1, and actin cytoplasmic 2 were over-expressed in the AUD group and the MCV-high AUD subgroup. Actin interacts with spectrin and band 4.1, underpinning structural integrity. Bulle et al. demonstrated that individuals with alcoholism exhibit a significant increase in plasma and erythrocyte membrane lipid peroxidation, as well as elevated levels of nitric oxide (NOx) in erythrocyte lysates. They proposed that heightened lipid and protein oxidation, changes in the membrane cholesterol-to-phospholipid ratio, and alterations in the membrane cytoskeletal protein profile may contribute to the increased hemolysis observed in alcoholics [48]. Additionally, SDS-PAGE analysis of erythrocyte membrane proteins in alcoholics revealed an increased density of band 3, protein 4.2, 4.9, actin, and glycophorins. Glyceraldehyde 3-phosphate dehydrogenase (GAPDH) and glycophorin A showed a slight increase, while ankyrin decreased. No significant changes were observed in spectrins (alpha and beta) and protein 4.1. Furthermore, erythrocytes from alcoholics exhibited altered morphology and decreased resistance to osmotic hemolysis [48].

Under oxidative stress, ATP synthesis declines due to the redox-sensitive nature of glycolytic enzymes and the band 3-dependent mechanism. This shift favors lower-energy phosphate compounds, such as ADP and AMP, with AMP being deaminated to IMP by erythrocyte-specific AMP deaminase 3. Moreover, AMP hydrolysis into adenosine and the subsequent oxidation by adenosine deaminase have been associated with hypoxanthine accumulation. The recycling of hypoxanthine via the X-linked hypoxanthine-guanine phosphoribosyltransferase (HPRT) enzyme plays a crucial role in maintaining the balance of inosine monophosphate (IMP) and guanosine monophosphate (GMP) within erythrocytes [49,50]. In this study, HPRT expression was upregulated in the MCV-high subgroup of AUD patients (Table 2), possibly as an adaptive or homeostatic response.

In a PTZ seizure mouse model for evaluation of epilepsy [51], the protein expression levels of thioredoxin-1 (TRX1), thioredoxin-like 1 protein (TXNL1), and thioredoxin reductase 1 (TXNRD1) were upregulated in the cortex of both acute and chronic groups. The authors have proposed that upregulation of these proteins may provide protection in the cortex during seizures while other antioxidants, including GSH, are suppressed. Txnl1 is a widely expressed, metabolically stable regulatory subunit of the human 26S proteasome, which is a large proteolytic machine, acting in the degradation of intracellular proteins. Txnl1/TRP32 may bind to Rpn11, a subunit of the regulatory complex of the human 26S proteasome. Moreover, Txnl1 has thioredoxin activity. In response to the knockdown of Txnl1, ubiquitin–protein conjugates were moderately stabilized. Therefore, Txnl1 represents the first instance of a direct link between protein degradation and proteolysis, two essential mechanisms in cellular protein quality control [52]. A study reported that in two patients with hereditary non-spherocytic hemolytic anemia, the expression levels of thioredoxin-like 1 protein were elevated compared to controls [53].

Among the upregulated proteins in the MCV-high group relative to the MCV-low group, alpha-synuclein emerges as a notable point of interest. This protein has been extensively researched for its involvement in Parkinson’s disease etiology and has been detected in blood samples. A targeted quantitative analysis indicated that alpha-synuclein predominantly resides within erythrocytes [54]. Higher levels of alpha-synuclein protein expression in plasma samples of AUD patients have been observed compared to controls; additionally, a significant link between protein expression levels and craving scores has been identified [55]. Presynaptic alpha-synuclein has been suggested to play a crucial role in regulating the dopamine system and the mechanisms of various addictions, including alcoholism. However, its specific function during different stages of addiction development remains unclear. The study by Anokhin et al. (2016) [56] investigated the expression of the alpha-synuclein gene in the brains of animals engaged in a “free-choice” alcohol consumption model. The findings revealed that animals with a high daily alcohol intake showed a significant decrease in alpha-synuclein expression in the midbrain and hypothalamus compared to animals with lower and more consistent alcohol consumption. Since these alterations were observed in brain regions involved in dopaminergic pathways, but not in areas targeted by dopamine neurons, this supports the hypothesis that alpha-synuclein may play a protective role in dopamine-producing brain regions, particularly in animals with a low alcohol preference, which can regulate their intake by maintaining stable alpha-synuclein levels [56]. The upregulation of 14-3-3 protein beta/alpha and 14-3-3 protein gamma in the erythrocytes of patients with polycythemia vera has been demonstrated in comparison to healthy individuals [57]. Furthermore, 14-3-3 proteins can be activated by stress and exhibit chaperone functions, particularly in the context of neurodegeneration. Their role in this context has garnered interest due to the presence of various isoforms in protein aggregates characteristic of neurodegenerative diseases, where they may modulate aggregation [58]. The connection between AUD and a heightened risk of developing Alzheimer’s and Parkinson’s diseases [59], along with the overlapping role of oxidative stress in AUD and Alzheimer’s disease [60], underscores the potential of this issue.

The downregulation of uroporphyrinogen decarboxylase in the MCV-high AUD subgroup in comparison with the MCV-normal AUD patients was another point of interest (Table 2). McColl et al. reported that ALA synthase activity, the initial and rate-limiting enzyme, was elevated, while the activities of ALA dehydratase and uroporphyrinogen decarboxylase were inhibited by alcohol. All enzyme activities returned to normal within 10–20 days of alcohol withdrawal. The authors suggest that shifts in enzyme activities may elucidate how long-term alcohol consumption induces cutaneous hepatic porphyria [61].

The functional annotation of erythrocyte cytosolic and membrane protein fractions in relation to differentially expressed proteins in erythrocyte samples of the study groups revealed that specific proteins are involved in the negative regulation of protein metabolic processes, catabolism, and responses to oxidative stress. Notably, we identified clusters related to modification-dependent protein catabolic processes, negative regulation of protein modification, regulation of cell death, ATP binding, and ubiquitin. Top hub genes/proteins obtained based on our assessments using cytoHubba methods included YWHAB/1433B, YWHAG/1433G, ACTG1/ACTG, HSP90B1/ENPL, YWHAE/1433E, YWHAZ/1433Z, ACTB, CAPZB, EIF2S1/IF2A, HSP90AA1/HS90A, HSPA8/HSP7C, EIF4A2/IF4A2, HSPA4/HSP74, SNCA/SYUA, and TPM3. According to these analyses, the key molecular functions related to the mentioned hubs include: the structural constituent of postsynaptic actin cytoskeleton, MHC class II protein complex binding, Tau protein binding, protein folding chaperone, phosphoserine residue binding, protein kinase inhibitor activity, ubiquitin protein ligase binding, cadherin binding, phosphoprotein binding, and unfolded protein binding. While key biological processes cover protein targeting, intracellular protein transport, regulation of norepinephrine uptake, response to unfolded protein, regulation of transepithelial transport, cellular response to heat, synaptic vesicle endocytosis, establishment of localization in cells, and chaperone-mediated autophagy.

Particularly noteworthy, based on current data, is that we identified several key considerations for recognizing hubs within this interactome. As illustrated in Figure 8 and Figure 10, utilizing multiple methods can be beneficial for obtaining complementary insights, consistent with the approach recommended in the original study [30]. While some algorithms produced nearly identical rankings, others may identify different hubs. To clarify these differences, we employed the Julius AI platform [31] to evaluate the results from twelve available algorithms and verified the findings:

- Degree, EPC, and Closeness are identical on top-15s, forming a cluster (Figure 9).
- MNC, MCC, Radiality, Stress, and Bottleneck are moderately similar to that cluster.
- “Betweenness vs. ClusteringCoefficient” is the most divergent pair. Additionally, clustering coefficient and EcCentricity are the most divergent, with the fewest shared proteins and weak edges.
- Among the hubs, YWHAG/1433G, YWHAB/1433B, YWHAZ/1433Z, YWHAE/1433E, ACTG1/ACTG, and HSP90B1/ENPL consistently appear across the top lists; ACTB, HSP90AA1/HS90A, and HSPA8/HSP7C are also frequent.
- The highest rank dispersion across algorithms appears in EIF5A/IF5A1 and EIF4A1/IF4A1.
- The primary deviations from the consensus in the algorithms are as follows: DMNC ranking HSP90AA1/HS90A significantly lower than other methods, and MCC assigning EIF5A/IF5A1 and HSPA4/HSP74 scores noticeably divergent from the consensus.

As previously mentioned, the common acceptance of erythrocytes as the simplest cell has been altered in view of studies underscoring their unique biology and specialized functions. A study on the “erythrocyte interactome” [62] has described the key points about the RBC interactome as follows: (1) It is remarkably compact, comprising just over 100 protein complexes. (2) The interactome is dominated by four major functional categories: proteostasis, cytoskeleton and structural integrity, energy production, and redox biology. (3) Proteins involved in proteostasis are prominent in the interactome, including the 19S regulatory cap and 20S core of the proteasome. However, these components are generally observed as distinct forms rather than assembled 26S proteasomes. (4) Energy production and redox-related protein complexes are abundant, suggesting a balance between energy production and management of toxic byproducts. (5) Cytoskeletal complexes, essential for maintaining RBC structural integrity and enabling cell morphology changes, are well-represented in the interactome. (6) The interactome reveals interactions between proteins involved in energy production and byproduct detoxification, such as the interaction between triosephosphate isomerase and the Parkinsonism-associated protein (DJ-1/Park7). Sae-Lee et al. (2022) [62] also underscore the molecular organization of erythrocytes, particularly the “band 3-ankyrin1” complex, which is crucial for maintaining the structural integrity and dynamic morphology. They suggest that this complex not only anchors the spectrin cytoskeleton to the membrane but also serves as a hub for metabolic processes, highlighting the intricate spatial organization of erythrocyte proteins that facilitates rapid morphological changes essential for their function in the circulatory system [62]. The study by Anastasiadi et al. (2021) [63] underscores the intricate behavior of proteasome activity in erythrocytes from β-thalassemia minor donors during storage. It reports a notable increase in membrane-bound proteasome activity alongside a decrease in cytosolic activity, linked to the cells’ lipid composition, structural features, and a “repair-or-destroy” protein network. These adaptations likely enhance oxidative stress management and proteostasis, suggesting that heightened membrane proteasome activity may serve a protective function, with implications for transfusion efficacy and clinical applications, especially in patients on proteasome inhibitors [63]. D’Alessandro et al. (2010) provide an updated and comprehensive view of the erythrocyte proteome and interactome, demonstrating the power of new proteomics and interactomics techniques in elucidating complex biological systems [64]. Their analysis emphasizes that integrated interactomics reveals intricate networks of protein interactions, essential for understanding erythrocyte function and pathology, especially in transfusion medicine and aging of stored blood components. Pathway analysis indicates increased oxidative stress in erythrocytes, consistent with their oxygen transport role. Network analysis further shows that the top-scoring networks are mainly devoted to protein protection against misfolding, with a potential “catalytic ring” of proteins enhancing the proposed “Repair or Destroy” (ROD) activity [64].

Although direct evidence linking alcohol to erythrocyte microparticle formation is lacking in the literature, a study involving sickle cell anemia patients indicated increased erythrocyte-derived microparticles associated with oxidative stress. Additionally, mechanistic studies in other cell types suggest that oxidative stress and phosphatidylserine externalization play roles in microparticle formation [65].

There are several limitations to our study. First, our research employed a modest sample size, which was nonetheless sufficient to generate meaningful and innovative insights into the subject matter. This approach provided a careful balance between depth and scope, enabling a comprehensive exploration of the key variables involved. The study was conducted exclusively with male participants due to sampling limitations. Therefore, further research is necessary to investigate potential gender differences in this area. Third, it is crucial that the proteins of interest are readily measurable within standard laboratory environments, and we are actively pursuing strategies to achieve this. While the preliminary results are promising, it is imperative to emphasize that validating these findings constitutes a critical endeavor. This necessity arises from the inherent limitations of our sample source and the extensive erythrocyte processing required. Addressing these concerns is essential to ensure the robustness and reliability of our conclusions. Therefore, despite encouraging findings, the validation of these results remains a necessary objective, given the current constraints.

The strength of this study lies in the comprehensive evaluation and comparison of protein expression alterations within erythrocyte cytosol and membrane fractions across three distinct groups: individuals with AUD, along with social drinkers, and non-drinking controls. Previous studies and current findings may have potential implications for understanding the pathophysiology of alcohol-related conditions, given that impaired erythrocyte functions may contribute to disrupted organ functions, which has been shown in hepatic microcirculation, where such impairments could exacerbate liver dysfunction in alcoholic patients [66]. The reduced aggregation and altered deformability could also influence cardiovascular risk; however, the overall clinical impact appears complicated. As reported previously, the reversibility of changes upon alcohol cessation offers hope for clinical improvement [67,68]; however, due to their character, some alterations might already be irreversible.

## 4. Materials and Methods

### 4.1. Materials

All chemicals were selected for proteomics applications whenever feasible; otherwise, they were of the highest available grade. The cytosolic and membrane protein fractions of erythrocyte samples were isolated using the ProteoJET Membrane Protein Extraction Kit from Fermentas (St. Leon-Rot, Germany), following the manufacturer’s protocol. Hemoglobin removal was performed with the HemoVoid kit (Biotech Support Group, Monmouth, NJ, USA). Ultrapure water (18.2 MΩ·cm), used consistently across all the experiments, was produced using a Direct-Q3 UV purification system (Merck Millipore, Darmstadt, Germany).

### 4.2. Sampling and Pre-Analytical Procedures

Blood samples were obtained from male participants categorized into three groups: individuals diagnosed with AUD (*n* = 30), social drinkers (SD, *n* = 15), and the control group (*n* = 15). The AUD group comprised individuals who met corresponding DSM-IV criteria and were recruited from the Alcohol and Drug Addiction Treatment and Research Center (AMATEM, Ankara, Türkiye). The control group, composed exclusively of individuals identified as “never drinkers” or “nondrinkers,” along with a cohort of social drinkers who consume alcohol occasionally, were matched to the AUD group based on age and body mass index (BMI). All the participants tested negative for hepatitis B and C antibodies. A comprehensive questionnaire was employed to collect data on the quantity and frequency of alcohol consumption, as well as additional relevant information regarding potential outcomes of alcohol use in individuals with AUD and social drinkers. In accordance with ethical guidelines and academic standards, the research protocol received approval from the Ethics Committee of Ankara Numune Hospital (01.02.2006/6), ensuring adherence to the Declaration of Helsinki. Informed consent was acquired from each participant following established guidelines and standards, and interviews were uniformly conducted at the study’s inception.

Blood samples were obtained in K_2_-EDTA tubes for proteomic investigations and in serum tubes for biochemical evaluations. After collection, samples were processed accordingly, aliquoted, and stored at −80 °C until subsequent analysis. Biochemical parameters were assessed by an accredited laboratory (Düzen Lab., Ankara, Türkiye). For the separation of the erythrocyte fraction, blood samples were initially centrifuged at 2500× *g* at 4 °C within 2 h of collection to separate plasma and the buffy coat. Subsequently, the erythrocyte fraction was prepared immediately. Briefly, each erythrocyte pellet was washed three times with phosphate-buffered saline, and then the cytosolic and membrane fractions were separated using the ProteoJET Membrane Protein Extraction Kit (Fermentas), following the manufacturer’s instructions. Since hemoglobin is the most common protein in erythrocytes, it was removed to improve the detection of low-abundance proteins. The HemoVoid kit from Biotech Support Group was used to assist this process. The resulting fractions were preserved at −80 °C for the subsequent steps. Total protein concentrations were quantified using the bichinchoninic acid (BCA) Protein Assay Kit from SIGMA (St. Louis, MO, USA), following standard procedures.

### 4.3. Two-Dimensional Gel Electrophoresis

The cytosolic and membrane fractions of erythrocytes were analyzed to assess variations in protein expression among individual patients. For each sample, 100 µg of protein was prepared using a rehydration buffer containing 7 M urea, 2 M thiourea, 0.5% ampholytes, 4% CHAPS, 20 mM DTT, and trace amounts of bromophenol blue. The mixtures were vortexed briefly, sonicated for 5–10 s to improve solubilization, and then centrifuged at 4 °C to eliminate any residual impurities.

In the initial dimension of separation, an Isoelectric Focusing (IEF) Cell (Bio-Rad Laboratories, Hercules, CA, USA) and 24 cm Immobiline DryStrip (IPG) strips with pH ranges of 3–10 and 4–7 (Bio-Rad) were utilized. Each sample, combined with the rehydration buffer, was meticulously pipetted into a focusing tray channel, followed by the rehydration of the IPG strips at 50 V for 12 h at 20 °C in the PROTEAN IEF Cell (Bio-Rad). The IEF procedure adhered to the manufacturer’s instructions, employing the following voltage stages: Stage 1: 0–500 V for 1 h; Stage 2: 1000 V for 1 h; Stage 3: 1000–10,000 V for 3 h; Stage 4: 10,000 V for 4 h. Upon completion of the IEF, the IPG strips were stored at −80 °C within sterile rehydration trays. Prior to SDS-PAGE, the IPG strips underwent sequential equilibration in two freshly prepared buffer solutions. The initial buffer contained 1% DTT to facilitate reduction, dissolved in 50 mM Tris-HCl (pH 8.8), 6 M urea, 30% glycerol, and 2% SDS. The subsequent buffer replaced DTT with 2.5% iodoacetamide (IAA) to achieve alkylation of cysteine residues and prevent the reformation of disulfide bonds. Each reduction and alkylation step lasted 15 min with gentle orbital shaking at room temperature, followed by rinsing with SDS-PAGE buffer, and subsequently each IPG strip was precisely positioned on a lab-cast 12.5% polyacrylamide gel prepared with the Ettan DALT Gel Caster, then sealed with 0.5% agarose solution. A vertical SDS-PAGE system (Ettan DALTsix, GE Healthcare, Uppsala, Sweden) was used, running for approximately six hours at 20 °C. Initially, a power of 1 W per gel was applied for the first hour, then increased to 13 W until the bromophenol blue dye migrated to the gel bottom. Protein standards were run with each gel (Unstained Precision Plus Protein Standard plugs, Bio-Rad).

After completing the electrophoresis, the 2D gels were first transferred to fixing solution comprising 10% methanol and 7% acetic acid for a 30 min fixing step, and then stained overnight. Prior to image acquisition, a wash step in the same fixing solution for 30 min at room temperature with gentle agitation, protected from light, was implemented to minimize background fluorescence. Gels imaged using ImageQuant 350, a 16-bit CCD camera (GE Healthcare, Piscataway, NJ, USA), were analyzed with SameSpots software v 4.0 (Nonlinear Dynamics, Newcastle-upon-Tyne, UK), and spots of interest were excised with a spot picker (GE Healthcare, Piscataway, NJ, USA).

### 4.4. MALDI-TOF MS and MALDI-TOF/TOF Tandem MS/MS

An AB SCIEX TOF/TOF 5800 System (AB SCIEX, Framingham, MA, USA) capable of MALDI-TOF MS and TOF/TOF tandem MS/MS was employed to identify the proteins by Applied Biomics, Inc. (Hayward, CA, USA). Briefly, the gel spots were subjected to multiple washes and digested in gel with modified porcine trypsin (Trypsin Gold, Promega, Madison, WI, USA). The tryptic peptides purified via Zip-tip C18 (Millipore, Billerica, MA, USA) and eluted with 0.5 µL of a matrix containing 5 mg/mL of α-cyano-4-hydroxycinnamic acid in a solution of 50% acetonitrile, 0.1% trifluoroacetic acid, and 25 mM ammonium bicarbonate were applied to a MALDI plate (Opti-TOF 384 Well Insert, AB SCIEX, Framingham, MA, USA) to collect MALDI-TOF mass spectra in reflectron positive ion mode, with each spectrum averaging 4000 laser shots. For each sample, TOF/TOF tandem MS spectra were collected by averaging 4000 laser shots on the ten most abundant ions, excluding trypsin autolytic peptides and background ions. Peptide mass data and fragmentation spectra were processed using the GPS Explorer (v3.6) workstation, integrated with the MASCOT search engine (Matrix Science, Boston, MA, USA), set for a Swiss-Prot database search. The search parameters permitted variable modifications such as carbamidomethylation, methionine oxidation, and one missed cleavage, without restrictions on protein MW or pI. Candidates with either a protein score confidence interval percentage (C.I.%) or an ion C.I.% above 95% were deemed highly confident.

### 4.5. Enrichment Analysis, Protein–Protein Interaction Network, and Selection of Hub Proteins

The DAVID Bioinformatics Database and shinyGO 0.82 were used for Gene Ontology (GO) enrichment analysis to determine the functions of the proteins of interest [26,69]. The constructed protein–protein interaction network was further visualized using the Cytoscape software v3.10.3 [70], and the cytoHubba [30] plugin, implemented in Java, 17.0.5 was employed to identify the top 15 hub proteins in the network. In this context, we applied all twelve available topological methods, including (1) Maximal Clique Centrality (MCC), (2) Density of Maximum Neighborhood Component (DMNC), (3) Maximum Neighborhood Component (MNC), (4) Degree, (5) Edge Percolated Component (EPC), (6) Bottleneck, (7) EcCentricity, (8) Closeness, (9) Radiality, (10) Betweenness, (11) Stress, and (12) ClusteringCoefficient, as listed in the order of appearance in the cytoHubba.

### 4.6. Serum %CDT, Total SA, and Biochemical Tests

A commercially available HPLC kit (%CDT, Bio-Rad Laboratories, München, Germany) and sialic acid kit (SA, Roche, Germany) were employed in accordance with the manufacturers’ instructions, as detailed previously [12]. Biochemical analyses, including serum MCV, GGT, AST, and ALT assays, were conducted by a certified laboratory (Düzen Laboratories, Ankara, Türkiye).

### 4.7. Statistical Analysis

The two-dimensional gel studies included spot detection, normalized volume estimation, and expression analysis, all performed with SameSpots v 4.0 (Nonlinear Dynamics, UK). Spots showing at least a 1.5 change and a *p*-value under 0.05 were deemed statistically significant. All other analyses were conducted using GraphPad Prism v10.5.0, with data shown as mean ± standard deviation. Sub-group comparisons were performed using one-way ANOVA, followed by Dunnett’s T3 post hoc test, to identify statistical differences across study groups, where a *p*-value < 0.05 indicates significance.

## 5. Conclusions

As outlined in previous studies [23,45], a comprehensive understanding of the intricate network of protein interactions within erythrocytes is essential. This knowledge can advance our insight into erythrocyte function and homeostasis, as well as enhance diagnostic and therapeutic approaches for related diseases or exposures. Understanding the underlying mechanisms is crucial for developing strategies to mitigate alcohol-related complications and for identifying biomarkers of alcohol-induced cellular damage. The complex interplay between oxidative stress, membrane composition, and cellular function represents a paradigm for understanding how chronic exposure affects cellular physiology. Furthermore, the influence of alcohol on erythrocyte microparticle formation and its potential connections to oxidative stress and other mechanisms involving protein expression alterations would be a valuable avenue for future academic research.

## Figures and Tables

**Table 1:** The characteristics and biochemical test results of the study groups.

Characteristic/Biochemical Test	Control (Non-Drinkers)	Social Drinkers	AUD-MCV Normal	AUD-MCV High
Participants (all male)	15	15	16	14
Age (years)	42.40 ± 8.33	43.53 ± 9.19	44.75 ± 9.60	44.14 ± 7.79
BMI (kg/m^2^)	27.35 ± 3.05	27.38 ± 1.87	25.20 ± 4.42	24.91 ± 3.46
Ethanol intake (grams of ethanol per day)	-	28.33 ± 8.38	203.0 ± 98.86 ^a,b^	232.0 ± 95.07 ^a,b^
Erythrocytes (M/mm^3^)	5.31 ± 0.30	5.45 ± 0.41	5.25 ± 0.48 ^e^	4.55 ± 0.43 ^a,b,d^
MCV (fL)	85.43 ± 2.64	85.68 ± 6.09	87.82 ± 3.20	96.77 ± 4.34
RDW (%)	12.84 ± 0.52	13.34 ± 1.29	14.33 ± 1.66	13.68 ± 1.33
Vitamin B_12_ (pg/mL)	242.6 ± 77.48	342.9 ± 149.0	443.4 ± 308.1 ^a,e^	590.0 ± 478.3 ^a,b,d^
Folic acid (ng/mL)	7.64 ± 2.05	9.54 ± 3.38	9.03 ± 2.70	6.64 ± 2.90
AST/ALT	0.88 ± 0.31	0.82 ± 0.18	1.35 ± 0.47	1.62 ± 0.56
GGT (IU/L)	28.00 ± 17.46	25.07 ± 5.95	117.9 ± 134.0 ^e^	331.6 ± 276.4 ^a,b,d^

All data represent mean ± standard deviation. ALT, alanine aminotransferase; AST, aspartate transaminase; BMI, body mass index; GGT, gamma-glutamyl transferase; MCV, mean corpuscular volume; RDW, red cell distribution width. ^a^ *p* < 0.05 as compared to control; ^b^ *p* < 0.05 as compared to SD; ^d^ *p* < 0.05 as compared to AUD-MCV-normal subgroup; ^e^ *p* < 0.05 as compared to AUD-MCV-high subgroup.

**Table 3:** Functional Enrichment—GO “Biological Process” (shinyGO 0.82, [28]).

FDR	nGenes	GO Terms or Pathways	Description
2.44 × 10^−5^	13	GO:0061024	Membrane organization
0.00069	26	GO:1901564	Organonitrogen compound metabolic process
0.00069	7	GO:1905475	Regulation of protein localization to the membrane
0.00089	6	GO:0007006	Mitochondrial membrane organization
0.00089	23	GO:0019538	Protein metabolic process
0.00089	4	GO:1900740	Positive regulation of protein insertion into the mitochondrial membrane involved in the apoptotic signaling pathway
0.00095	18	GO:0032268	Regulation of the cellular protein metabolic process
0.00095	21	GO:0044267	Cellular protein metabolic process
0.00095	17	GO:0051649	Establishment of localization in cells
0.00095	22	GO:0065008	Regulation of biological quality
0.0020	22	GO:0006810	Transport
0.0024	18	GO:0051641	Cellular localization
0.0028	4	GO:0030834	Regulation of actin filament depolymerization
0.0029	5	GO:0043244	Regulation of protein-containing complex disassembly
0.0032	6	GO:0006839	Mitochondrial transport
0.0033	7	GO:0006605	Protein targeting
0.0033	19	GO:0006996	Organelle organization
0.0033	24	GO:0016043	Cellular component organization
0.0033	8	GO:0030036	Actin cytoskeleton organization
0.0034	27	GO:0006807	Nitrogen compound metabolic process
0.0034	7	GO:1902903	Regulation of supramolecular fiber organization
0.0043	5	GO:1902904	Negative regulation of supramolecular fiber organization
0.0051	8	GO:1903827	Regulation of cellular protein localization
0.0052	4	GO:0032272	Negative regulation of protein polymerization
0.0070	5	GO:0006986	Response to unfolded protein
0.0071	10	GO:0060341	Regulation of cellular localization
0.0073	13	GO:0016192	Vesicle-mediated transport
0.0073	6	GO:0031647	Regulation of protein stability
0.0073	22	GO:0044260	Cellular macromolecule metabolic process
0.0073	15	GO:0051128	Regulation of cellular component organization

**Table 4:** Functional Enrichment—GO “Cellular Component” (shinyGO, 0.82, [28]).

FDR	nGenes	GO Terms or Pathways	Description
1.06 × 10^−12^	34	GO:0005829	Cytosol
1.06 × 10^−12^	25	GO:0070062	Extracellular exosome
4.41 × 10^−10^	26	GO:0005615	Extracellular space
2.91 × 10^−7^	38	GO:0005737	Cytoplasm
6.17 × 10^−7^	7	GO:0072562	Blood microparticle
7.68 × 10^−7^	10	GO:0005925	Focal adhesion
9.19 × 10^−6^	6	GO:0042470	Melanosome
3.8 × 10^−5^	11	GO:0070161	Anchoring junction
0.00016	5	GO:0030863	Cortical cytoskeleton
0.00036	8	GO:0015629	Actin cytoskeleton
0.00079	36	GO:0043227	Membrane-bounded organelle
0.0013	37	GO:0043226	Organelle
0.0018	6	GO:0005938	Cell cortex
0.0024	8	GO:0030424	Axon
0.0024	22	GO:0032991	Protein-containing complex
0.0024	6	GO:0150034	Distal axon
0.0048	8	GO:0048471	Perinuclear region of cytoplasm
0.0062	13	GO:0030054	Cell junction
0.0066	2	GO:0097433	Dense body
0.0070	3	GO:0043209	Myelin sheath
0.0070	4	GO:1904813	ficolin-1-rich granule lumen
0.0103	13	GO:0005856	Cytoskeleton
0.0180	2	GO:0016281	Eukaryotic translation initiation factor 4F complex
0.0186	3	GO:0030864	Cortical actin cytoskeleton
0.0208	34	GO:0043229	Intracellular organelle
0.0218	25	GO:0005634	Nucleus
0.040	2	GO:0071682	Endocytic vesicle lumen
0.0478	4	GO:0030016	Myofibril
0.0491	3	GO:0005884	Actin filament

**Table 5:** Functional Enrichment—GO “Molecular Function” (shinyGO 0.82, [28]).

FDR	nGenes	GO Terms or Pathways	Description
0.00025	30	GO:0005515	Protein binding
0.00043	13	GO:0044877	Protein-containing complex binding
0.0015	11	GO:0008092	Cytoskeletal protein binding
0.0015	5	GO:0032182	Ubiquitin-like protein binding
0.0015	4	GO:0048156	Tau protein binding
0.0034	3	GO:0023026	MHC class II protein complex binding
0.0034	14	GO:0042802	Identical protein binding
0.0073	4	GO:0043130	Ubiquitin binding
0.0077	4	GO:0008135	Translation factor activity, RNA binding
0.0077	6	GO:0031625	Ubiquitin protein ligase binding
0.011	12	GO:0003723	RNA binding
0.011	6	GO:0045296	Cadherin binding
0.011	5	GO:0051015	Actin filament binding
0.0145	2	GO:0098973	Structural constituent of postsynaptic actin cytoskeleton
0.0251	3	GO:0003743	Translation initiation factor activity
0.0264	2	GO:0050815	Phosphoserine residue binding
0.03	6	GO:0003779	Actin binding
0.03	13	GO:0019899	Enzyme binding

## Data Availability

The raw data supporting the conclusions of this article will be made available by the authors on reques.

## Supplementary Materials

The following supporting information can be downloaded at: https://www.mdpi.com/article/10.3390/ijms26178199/s1.

## Abbreviations

The following abbreviations are used in this manuscript:

%CDT	Carbohydrate-deficient transferrin
%DST	Disialotransferrin
2,3-BPG	2,3-bisphosphoglycerate
2D	Two-dimensional
2D-GE	Two-dimensional Gel Electrophoresis
3D	Three-dimensional
ACTB	Actin, cytoplasmic 1
ACTG1/ACTG	Actin, cytoplasmic 2
ALT	Alanine aminotransferase
ANOVA	Analysis of variance
APEH/ACPH	Acylamino-acid-releasing enzyme
AST	Aspartate transaminase
AUD	Alcohol use disorders
BCA	Bicinchoninic acid
BPGM/PMGE	Bisphosphoglycerate mutase
CAPZB	F-actin-capping protein subunit beta
CLIC1	Chloride intracellular channel protein 1
DCUN1D1/DCNL1	DCN1-like protein 1
DDI2	Protein DDI1 homolog 2
DTT	Dithiothreitol
EIF2S1/IF2A	Eukaryotic translation initiation factor 2 subunit 1
EIF4A1/IF4A1	Eukaryotic initiation factor 4A-I
EIF4A2/IF4A2	Eukaryotic initiation factor 4A-II
EIF5A/IF5A1	Eukaryotic translation initiation factor 5A-1
GAPDH	Glyceraldehyde 3-phosphate dehydrogenase
GDI1/GDIA	Rab GDP dissociation inhibitor alpha
GGT	Gamma-glutamyl transferase
GO	Gene Ontology
GSH	Reduced glutathione
GSN/GELS	Gelsolin
GSSG	Oxidized glutathione
HAE	4-Hydroxyalkenals
HPRT1/HPRT	Hypoxanthine-guanine phosphoribosyltransferase
HSP90AA1/HS90A	Heat shock protein HSP 90-alpha
HSP90B1/ENPL	Endoplasmin
HSPA4/HSP74	Heat shock 70 kDa protein 4
HSPA8/HSP7C	Heat shock cognate 71 kDa protein
IAA	Iodoacetamide
ICD-11	International Classification of Diseases 11th Revision
IEF	Isoelectric Focusing
IMP	Inosine monophosphate
IPG	Immobiline™ pH gradient
MALDI-TOF/TOF	Matrix-Assisted Laser Desorption/Ionization Time-of-flight (TOF) mass spectrometry
MDA	Malondialdehyde
MW	Molecular weight
NOx	Nitric oxide
NUDT5	ADP-sugar pyrophosphatase
OSI	Oxidative stress index
OTUB1	Ubiquitin thioesterase
pI	Isoelectric point
PPP1R7/PP1R7	Protein phosphatase 1 regulatory subunit 7
PPP2CB/PP2AB	Serine/threonine-protein phosphatase 2A catalytic subunit beta isoform
RAD23A/RD23A	UV excision repair protein RAD23 homolog A
RDW	Red cell distribution width
ROC	Receiver operating characteristic
SDS-PAGE	Sodium Dodecyl Sulfate Polyacrylamide Gel Electrophoresis
SLC4A1/B3AT	Band 3 anion transport protein
SNCA/SYUA	Alpha-synuclein
SPTA1	Spectrin alpha chain, erythrocyte
TAC	Total antioxidant capacity
TGM2	Protein-glutamine gamma-glutamyltransferase 2
TOS	Total oxidative status
TPM3	Tropomyosin alpha-3 chain
TRX1	Thioredoxin-1
TTLL12/TTL12	Tubulin–tyrosine ligase-like protein 12
TXNL1	Thioredoxin-like protein 1
TXNRD1	Thioredoxin reductase 1
UROD/DCUP	Uroporphyrinogen decarboxylase
USP5/UBP5	Ubiquitin carboxyl-terminal hydrolase 5
WDR1	WD repeat-containing protein 1
YWHAB/1433B	14-3-3 protein beta/alpha
YWHAE/1433E	14-3-3 protein epsilon
YWHAG/1433G	14-3-3 protein gamma
YWHAZ/1433Z	14-3-3 protein zeta/delta
