# Supplementary material for: Differential Expression of Erythrocyte Proteins in Patients with Alcohol Use Disorder
Source: Int J Mol Sci. 2025 Aug 23;26(17):8199. doi: 10.3390/ijms26178199 (PMC12428514; doi:10.3390/ijms26178199)
Supplement: Supplementary file 1 [file ijms-26-08199-s001.zip › Table S2.pdf]

Functional Annotation Clustering

Help and Manual

Current Gene List: List_1
Current Background: Homo sapiens
38 DAVID IDs

Options **Classification Stringency** Medium

Rerun using options | Create Sublist

9 Cluster(s)

Download File

			Count	P_Value	Fold Change	Bonferroni	Benjamini	FDR
Annotation Cluster 1	Enrichment Score: 4.24	G	Count	P_Value	Fold Change	Bonferroni	Benjamini	FDR
GOTERM_CC_DIRECT	focal adhesion	RT	10	5.0E-8	1.3E1	7.0E-6	2.3E-6	2.1E-6
UP_SEQ_FEATURE	DOMAIN:14-3-3	RT	4	1.1E-7	3.6E2	3.6E-5	3.2E-5	3.2E-5
INTERPRO	14-3-3	RT	4	1.8E-7	3.1E2	2.9E-5	7.3E-6	7.0E-6
INTERPRO	14-3-3_CS	RT	4	1.8E-7	3.1E2	2.9E-5	7.3E-6	7.0E-6
INTERPRO	14-3-3_domain	RT	4	1.8E-7	3.1E2	2.9E-5	7.3E-6	7.0E-6
INTERPRO	14-3-3_dom_sf	RT	4	1.8E-7	3.1E2	2.9E-5	7.3E-6	7.0E-6
UP_SEQ_FEATURE	SITE:Interaction with phosphoserine on interacting protein	RT	4	1.8E-7	3.1E2	6.3E-5	3.2E-5	3.2E-5
SMART	14_3_3	RT	4	2.3E-7	2.8E2	5.0E-6	5.0E-6	5.0E-6
GOTERM_CC_DIRECT	melanosome	RT	6	9.9E-7	3.2E1	1.4E-4	2.3E-5	2.0E-5
KEGG_PATHWAY	Hippo signaling pathway	RT	7	2.1E-6	1.7E1	1.9E-4	1.9E-4	1.7E-4
PIR_SUPERFAMILY	14-3-3	RT	4	3.4E-6	1.0E2	2.4E-5	2.4E-5	2.4E-5
KEGG_PATHWAY	Hepatitis C	RT	6	4.3E-5	1.4E1	4.0E-3	2.0E-3	1.7E-3
GOTERM_MF_DIRECT	protein sequestering activity	RT	4	5.3E-5	5.3E1	9.6E-3	2.5E-3	2.2E-3
GOTERM_MF_DIRECT	phosphoserine residue binding	RT	3	5.4E-5	2.5E2	9.8E-3	2.5E-3	2.2E-3
GOTERM_BP_DIRECT	protein targeting	RT	4	7.3E-5	4.8E1	3.3E-2	3.4E-2	3.4E-2
KEGG_PATHWAY	PI3K-Akt signaling pathway	RT	7	2.3E-4	7.2E0	2.2E-2	7.3E-3	6.3E-3
GOTERM_MF_DIRECT	ubiquitin protein ligase binding	RT	6	2.7E-4	1.0E1	4.9E-2	7.2E-3	6.4E-3
GOTERM_MF_DIRECT	cadherin binding	RT	6	3.4E-4	9.5E0	6.1E-2	7.5E-3	6.7E-3
KEGG_PATHWAY	Oocyte meiosis	RT	5	3.9E-4	1.3E1	3.6E-2	9.1E-3	7.9E-3
KEGG_PATHWAY	Cell cycle	RT	5	6.4E-4	1.2E1	5.8E-2	1.2E-2	1.0E-2
GOTERM_MF_DIRECT	MHC class II protein complex binding	RT	3	1.2E-3	5.6E1	2.0E-1	1.8E-2	1.6E-2
KEGG_PATHWAY	Viral carcinogenesis	RT	5	1.7E-3	9.0E0	1.5E-1	2.2E-2	1.9E-2
GOTERM_MF_DIRECT	transmembrane transporter binding	RT	4	2.6E-3	1.4E1	3.9E-1	2.9E-2	2.5E-2
GOTERM_BP_DIRECT	protein localization	RT	4	3.2E-3	1.3E1	7.8E-1	2.2E-1	2.2E-1
GOTERM_MF_DIRECT	protein phosphatase inhibitor activity	RT	3	3.8E-3	3.2E1	5.1E-1	3.7E-2	3.3E-2
GOTERM_MF_DIRECT	protein domain specific binding	RT	4	8.3E-3	9.3E0	7.8E-1	6.6E-2	5.9E-2
GOTERM_MF_DIRECT	protein phosphatase binding	RT	3	1.2E-2	1.7E1	9.0E-1	9.5E-2	8.5E-2
GOTERM_MF_DIRECT	histone deacetylase binding	RT	3	2.5E-2	1.2E1	9.9E-1	1.7E-1	1.5E-1
GOTERM_BP_DIRECT	signal transduction	RT	7	3.1E-2	2.8E0	1.0E0	5.2E-1	5.2E-1
GOTERM_MF_DIRECT	enzyme binding	RT	3	1.6E-1	4.1E0	1.0E0	8.3E-1	7.4E-1
Annotation Cluster 2	Enrichment Score: 2.69	G	Count	P_Value	Fold Change	Bonferroni	Benjamini	FDR
GOTERM_MF_DIRECT	ubiquitin binding	RT	4	9.3E-4	2.0E1	1.6E-1	1.6E-2	1.4E-2
SMART	UBA	RT	3	1.5E-3	4.9E1	3.3E-2	1.7E-2	1.7E-2
INTERPRO	UBA	RT	3	2.9E-3	3.7E1	3.7E-1	7.8E-2	7.5E-2
UP_SEQ_FEATURE	DOMAIN:UBA	RT	3	4.2E-3	3.0E1	7.6E-1	2.4E-1	2.4E-1
Annotation Cluster 3	Enrichment Score: 2.5	G	Count	P_Value	Fold Change	Bonferroni	Benjamini	FDR
GOTERM_CC_DIRECT	melanosome	RT	6	9.9E-7	3.2E1	1.4E-4	2.3E-5	2.0E-5
GOTERM_MF_DIRECT	ATP-dependent protein folding chaperone	RT	4	7.6E-5	4.7E1	1.4E-2	2.5E-3	2.2E-3
KEGG_PATHWAY	Protein processing in endoplasmic reticulum	RT	5	8.4E-4	1.1E1	7.5E-2	1.3E-2	1.1E-2
GOTERM_MF_DIRECT	ATP hydrolysis activity	RT	6	1.3E-3	7.0E0	2.2E-1	1.8E-2	1.6E-2
UP_KW_BIOLOGICAL_PROCESS	Stress response	RT	4	1.4E-3	1.7E1	3.5E-2	1.8E-2	1.8E-2
KEGG_PATHWAY	Lipid and atherosclerosis	RT	5	2.0E-3	8.6E0	1.7E-1	2.4E-2	2.1E-2
GOTERM_MF_DIRECT	ATP binding	RT	10	2.1E-3	3.3E0	3.3E-1	2.6E-2	2.3E-2
GOTERM_BP_DIRECT	protein folding	RT	4	4.2E-3	1.2E1	8.5E-1	2.2E-1	2.2E-1
KEGG_PATHWAY	Fluid shear stress and atherosclerosis	RT	4	5.4E-3	1.1E1	4.0E-1	5.6E-2	4.9E-2
UP_KW_LIGAND	Nucleotide-binding	RT	11	5.7E-3	2.3E0	5.5E-2	5.7E-2	5.7E-2
GOTERM_BP_DIRECT	response to unfolded protein	RT	3	6.4E-3	2.4E1	9.5E-1	2.4E-1	2.4E-1
UP_KW_LIGAND	ATP-binding	RT	9	1.4E-2	2.4E0	1.3E-1	6.9E-2	6.9E-2

Category	Term	RT	Count	P_Value	Fold Change	Bonferroni	Benjamini	FDR
Annotation Cluster 1	Enrichment Score: 4.24	G	Count	P_Value	Fold Change	Bonferroni	Benjamini	FDR
KEGG_PATHWAY	Antigen processing and presentation	RT	3	1.8E-2	1.4E1	8.2E-1	1.4E-1	1.2E-1
GOTERM_MF_DIRECT	unfolded protein binding	RT	3	2.5E-2	1.2E1	9.9E-1	1.7E-1	1.5E-1
KEGG_PATHWAY	Salmonella infection	RT	4	2.6E-2	5.9E0	9.1E-1	1.6E-1	1.4E-1
KEGG_PATHWAY	Estrogen signaling pathway	RT	3	4.9E-2	8.0E0	9.9E-1	2.5E-1	2.2E-1
UP_KW_MOLECULAR_FUNCTION	Chaperone	RT	3	1.1E-1	5.2E0	9.6E-1	4.9E-1	4.7E-1
Annotation Cluster 4	Enrichment Score: 2.29	G	Count	P_Value	Fold Change	Bonferroni	Benjamini	FDR
GOTERM_CC_DIRECT	actin cytoskeleton	RT	8	7.0E-7	1.5E1	9.8E-5	2.0E-5	1.7E-5
GOTERM_MF_DIRECT	actin filament binding	RT	5	7.7E-4	1.2E1	1.3E-1	1.4E-2	1.3E-2
UP_KW_MOLECULAR_FUNCTION	Actin capping	RT	3	8.2E-4	6.7E1	2.3E-2	1.1E-2	1.1E-2
GOTERM_MF_DIRECT	structural constituent of cytoskeleton	RT	4	1.3E-3	1.8E1	2.2E-1	1.8E-2	1.6E-2
UP_KW_MOLECULAR_FUNCTION	Actin-binding	RT	5	4.0E-3	7.3E0	1.1E-1	3.8E-2	3.6E-2
GOTERM_MF_DIRECT	actin binding	RT	5	4.4E-3	7.3E0	5.5E-1	4.0E-2	3.6E-2
UP_KW_CELLULAR_COMPONENT	Cytoskeleton	RT	9	4.6E-3	3.2E0	9.3E-2	4.9E-2	4.6E-2
GOTERM_CC_DIRECT	cortical actin cytoskeleton	RT	3	4.8E-3	2.8E1	4.9E-1	4.9E-2	4.3E-2
KEGG_PATHWAY	Motor proteins	RT	4	1.4E-2	7.5E0	7.2E-1	1.1E-1	9.9E-2
GOTERM_CC_DIRECT	actin filament	RT	3	1.4E-2	1.6E1	8.5E-1	1.1E-1	9.5E-2
GOTERM_CC_DIRECT	Schaffer collateral - CA1 synapse	RT	3	1.4E-2	1.6E1	8.6E-1	1.1E-1	9.5E-2
GOTERM_CC_DIRECT	cytoskeleton	RT	5	2.1E-2	4.6E0	9.5E-1	1.4E-1	1.2E-1
KEGG_PATHWAY	Cytoskeleton in muscle cells	RT	4	2.1E-2	6.4E0	8.6E-1	1.5E-1	1.3E-1
KEGG_PATHWAY	Hypertrophic cardiomyopathy	RT	3	2.6E-2	1.1E1	9.2E-1	1.6E-1	1.4E-1
GOTERM_BP_DIRECT	regulation of cell shape	RT	3	2.6E-2	1.2E1	1.0E0	5.1E-1	5.1E-1
GOTERM_BP_DIRECT	actin filament organization	RT	3	2.9E-2	1.1E1	1.0E0	5.2E-1	5.2E-1
KEGG_PATHWAY	Dilated cardiomyopathy	RT	3	3.0E-2	1.1E1	9.4E-1	1.7E-1	1.5E-1
GOTERM_BP_DIRECT	cytoskeleton organization	RT	3	3.4E-2	1.0E1	1.0E0	5.2E-1	5.2E-1
Annotation Cluster 5	Enrichment Score: 2.17	G	Count	P_Value	Fold Change	Bonferroni	Benjamini	FDR
GOTERM_CC_DIRECT	blood microparticle	RT	7	2.3E-7	2.6E1	3.2E-5	8.0E-6	7.0E-6
GOTERM_BP_DIRECT	platelet aggregation	RT	3	3.7E-3	3.2E1	8.2E-1	2.2E-1	2.2E-1
GOTERM_CC_DIRECT	vesicle	RT	3	3.4E-2	1.0E1	9.9E-1	2.0E-1	1.8E-1
GOTERM_CC_DIRECT	extracellular space	RT	7	1.1E-1	2.1E0	1.0E0	3.7E-1	3.2E-1
KEGG_PATHWAY	Regulation of actin cytoskeleton	RT	3	1.2E-1	4.8E0	1.0E0	5.5E-1	4.8E-1
GOTERM_MF_DIRECT	protein kinase binding	RT	3	2.5E-1	3.1E0	1.0E0	1.0E0	9.0E-1
Annotation Cluster 6	Enrichment Score: 2.13	G	Count	P_Value	Fold Change	Bonferroni	Benjamini	FDR
BIOCARTA	Eukaryotic protein translation	RT	3	3.2E-3	3.0E1	7.9E-2	7.9E-2	7.9E-2
UP_KW_BIOLOGICAL_PROCESS	Protein biosynthesis	RT	4	3.3E-3	1.3E1	7.9E-2	2.7E-2	2.7E-2
GOTERM_BP_DIRECT	translational initiation	RT	3	4.5E-3	2.9E1	8.7E-1	2.2E-1	2.2E-1
GOTERM_MF_DIRECT	translation initiation factor activity	RT	3	5.7E-3	2.6E1	6.5E-1	4.8E-2	4.3E-2
UP_KW_MOLECULAR_FUNCTION	Initiation factor	RT	3	7.0E-3	2.3E1	1.8E-1	4.9E-2	4.7E-2
UP_KW_MOLECULAR_FUNCTION	RNA-binding	RT	5	8.9E-2	2.8E0	9.3E-1	4.9E-1	4.7E-1
Annotation Cluster 7	Enrichment Score: 2.04	G	Count	P_Value	Fold Change	Bonferroni	Benjamini	FDR
INTERPRO	ATPase_NBD	RT	4	3.2E-4	2.9E1	5.0E-2	1.0E-2	9.9E-3
KEGG_PATHWAY	Tight junction	RT	4	9.1E-3	8.7E0	5.7E-1	8.4E-2	7.3E-2
UP_KW_PTM	Methylation	RT	5	2.6E-1	1.9E0	9.9E-1	5.9E-1	5.1E-1
Annotation Cluster 8	Enrichment Score: 1.72	G	Count	P_Value	Fold Change	Bonferroni	Benjamini	FDR
GOTERM_MF_DIRECT	ubiquitin binding	RT	4	9.3E-4	2.0E1	1.6E-1	1.6E-2	1.4E-2
GOTERM_BP_DIRECT	proteolysis	RT	5	2.6E-2	4.3E0	1.0E0	5.1E-1	5.1E-1
INTERPRO	Papain-like_cys_pep_sf	RT	3	3.9E-2	9.3E0	1.0E0	3.2E-1	3.1E-1
UP_KW_MOLECULAR_FUNCTION	Protease	RT	4	1.4E-1	2.9E0	9.9E-1	5.7E-1	5.5E-1
Annotation Cluster 9	Enrichment Score: 1.06	G	Count	P_Value	Fold Change	Bonferroni	Benjamini	FDR
KEGG_PATHWAY	Apoptosis	RT	3	4.7E-2	8.2E0	9.9E-1	2.5E-1	2.2E-1
GOTERM_CC_DIRECT	synapse	RT	4	6.7E-2	4.1E0	1.0E0	2.8E-1	2.4E-1
KEGG_PATHWAY	Influenza A	RT	3	7.2E-2	6.4E0	1.0E0	3.5E-1	3.1E-1
KEGG_PATHWAY	Amyotrophic lateral sclerosis	RT	3	2.5E-1	3.0E0	1.0E0	7.7E-1	6.7E-1

81 terms were not clustered.

Please cite DAVID within any publication that makes use of any methods inspired by **DAVID**.

Terms of Service | Contact Us | HHS Vulnerability Disclosure | Accessibility | Freedom of Information Act | Privacy Policy | No Fear Act | Office of Inspector General | USA.gov | Disclaimers
