# Supplementary material for: Differential Expression of Erythrocyte Proteins in Patients with Alcohol Use Disorder
Source: Int J Mol Sci. 2025 Aug 23;26(17):8199. doi: 10.3390/ijms26178199 (PMC12428514; doi:10.3390/ijms26178199)
Supplement: Supplementary file 1 [file ijms-26-08199-s001.zip › Table S1.pdf]

Functional Annotation Chart

[Help and Manual](#)

Current Gene List: List_1
Current Background: Homo sapiens
38 DAVID IDs

Options

Rerun Using Options | Create Sublist

125 chart records

Download File

Sublist	Category	Term	RT	Genes	Count	%	P-Value	Fold Enrichment	Bonferroni	Benjamini	FDR	Fisher Exact
	GOTERM_CC_DIRECT	cytosol	RT		35	92,1	2,3E-16	3,4	3,1E-14	3,3E-14	2,9E-14	6,9E-17
	GOTERM_CC_DIRECT	extracellular exosome	RT		25	65,8	4,8E-15	6,1	6,7E-13	3,4E-13	3,0E-13	7,3E-16
	UP_KW_PTM	Acetylation	RT		30	78,9	6,1E-13	3,4	9,1E-12	9,8E-12	8,5E-12	1,8E-13
	UP_KW_CELLULAR_COMPONENT	Cytoplasm	RT		31	81,6	2,4E-11	2,7	5,1E-10	5,1E-10	4,8E-10	8,9E-12
	SMART	14_3_3	RT		4	10,5	2,3E-7	278,1	5,0E-6	5,0E-6	5,0E-6	4,7E-10
	GOTERM_CC_DIRECT	focal adhesion	RT		10	26,3	5,0E-8	12,6	7,0E-6	2,3E-6	2,1E-6	3,9E-9
	PIR_SUPERFAMILY	14-3-3	RT		4	10,5	3,4E-6	100,8	2,4E-5	2,4E-5	2,4E-5	1,9E-8
	INTERPRO	14-3-3_dom_sf	RT		4	10,5	1,8E-7	312,9	2,9E-5	7,3E-6	7,0E-6	3,3E-10
	INTERPRO	14-3-3_CS	RT		4	10,5	1,8E-7	312,9	2,9E-5	7,3E-6	7,0E-6	3,3E-10
	INTERPRO	14-3-3_domain	RT		4	10,5	1,8E-7	312,9	2,9E-5	7,3E-6	7,0E-6	3,3E-10
	INTERPRO	14-3-3	RT		4	10,5	1,8E-7	312,9	2,9E-5	7,3E-6	7,0E-6	3,3E-10
	GOTERM_CC_DIRECT	blood microparticle	RT		7	18,4	2,3E-7	25,9	3,2E-5	8,0E-6	7,0E-6	8,4E-9
	UP_SEQ_FEATURE	DOMAIN:14-3-3	RT		4	10,5	1,1E-7	362,7	3,6E-5	3,2E-5	3,2E-5	1,5E-10
	UP_SEQ_FEATURE	SITE:Interaction with phosphoserine on interacting protein	RT		4	10,5	1,8E-7	310,9	6,3E-5	3,2E-5	3,2E-5	3,4E-10
	GOTERM_CC_DIRECT	actin cytoskeleton	RT		8	21,1	7,0E-7	15,0	9,8E-5	2,0E-5	1,7E-5	4,5E-8
	GOTERM_CC_DIRECT	melanosome	RT		6	15,8	9,9E-7	32,2	1,4E-4	2,3E-5	2,0E-5	2,9E-8
	KEGG_PATHWAY	Hippo signaling pathway	RT		7	18,4	2,1E-6	16,5	1,9E-4	1,9E-4	1,7E-4	1,2E-7
	UP_KW_MOLECULAR_FUNCTION	Hydrolase	RT		14	36,8	7,4E-5	3,2	2,1E-3	2,1E-3	2,0E-3	2,3E-5
	KEGG_PATHWAY	Hepatitis C	RT		6	15,8	4,3E-5	14,0	4,0E-3	2,0E-3	1,7E-3	3,0E-6
	GOTERM_CC_DIRECT	nucleus	RT		24	63,2	5,1E-5	2,1	7,2E-3	1,0E-3	9,0E-4	2,4E-5
	UP_KW_BIOLOGICAL_PROCESS	Host-virus interaction	RT		8	21,1	2,9E-4	5,5	7,3E-3	7,4E-3	7,4E-3	5,2E-5
	UP_KW_PTM	Phosphoprotein	RT		31	81,6	6,1E-4	1,5	9,2E-3	4,9E-3	4,3E-3	4,2E-4
	GOTERM_MF_DIRECT	protein binding	RT		37	97,4	5,3E-5	1,4	9,6E-3	2,5E-3	2,2E-3	3,8E-5
	GOTERM_MF_DIRECT	protein sequestering activity	RT		4	10,5	5,3E-5	53,3	9,6E-3	2,5E-3	2,2E-3	9,1E-7
	GOTERM_MF_DIRECT	phosphoserine residue binding	RT		3	7,9	5,4E-5	253,3	9,8E-3	2,5E-3	2,2E-3	1,4E-7
	GOTERM_MF_DIRECT	identical protein binding	RT		13	34,2	7,6E-5	3,7	1,4E-2	2,5E-3	2,2E-3	2,0E-5
	GOTERM_MF_DIRECT	ATP-dependent protein folding chaperone	RT		4	10,5	7,6E-5	47,1	1,4E-2	2,5E-3	2,2E-3	1,5E-6
	GOTERM_CC_DIRECT	cytoplasm	RT		23	60,5	1,0E-4	2,1	1,4E-2	1,8E-3	1,6E-3	4,7E-5
	GOTERM_MF_DIRECT	tau protein binding	RT		4	10,5	8,2E-5	46,1	1,5E-2	2,5E-3	2,2E-3	1,7E-6
	KEGG_PATHWAY	PI3K-Akt signaling pathway	RT		7	18,4	2,3E-4	7,2	2,2E-2	7,3E-3	6,3E-3	3,2E-5
	UP_KW_MOLECULAR_FUNCTION	Actin capping	RT		3	7,9	8,2E-4	67,4	2,3E-2	1,1E-2	1,1E-2	1,1E-5
	GOTERM_BP_DIRECT	protein targeting	RT		4	10,5	7,3E-5	47,9	3,3E-2	3,4E-2	3,4E-2	1,4E-6
	SMART	UBA	RT		3	7,9	1,5E-3	48,7	3,3E-2	1,7E-2	1,7E-2	2,9E-5
	UP_KW_BIOLOGICAL_PROCESS	Stress response	RT		4	10,5	1,4E-3	17,0	3,5E-2	1,8E-2	1,8E-2	8,0E-5
	KEGG_PATHWAY	Oocyte meiosis	RT		5	13,2	3,9E-4	13,3	3,6E-2	9,1E-3	7,9E-3	2,8E-5
	GOTERM_CC_DIRECT	perinuclear region of cytoplasm	RT		8	21,1	3,1E-4	5,8	4,2E-2	4,8E-3	4,2E-3	5,2E-5
	GOTERM_MF_DIRECT	ubiquitin protein ligase binding	RT		6	15,8	2,7E-4	10,0	4,9E-2	7,2E-3	6,4E-3	2,7E-5
	INTERPRO	ATPase_NBD	RT		4	10,5	3,2E-4	29,2	5,0E-2	1,0E-2	9,9E-3	1,0E-5
	UP_KW_LIGAND	Nucleotide-binding	RT		11	28,9	5,7E-3	2,3	5,5E-2	5,7E-2	5,7E-2	2,4E-3
	KEGG_PATHWAY	Cell cycle	RT		5	13,2	6,4E-4	11,7	5,8E-2	1,2E-2	1,0E-2	5,2E-5
	GOTERM_MF_DIRECT	cadherin binding	RT		6	15,8	3,4E-4	9,5	6,1E-2	7,5E-3	6,7E-3	3,5E-5
	GOTERM_MF_DIRECT	RNA binding	RT		11	28,9	3,7E-4	3,8	6,5E-2	7,5E-3	6,7E-3	9,5E-5
	KEGG_PATHWAY	Protein processing in endoplasmic reticulum	RT		5	13,2	8,4E-4	10,9	7,5E-2	1,3E-2	1,1E-2	7,4E-5
	BIOCARTA	Eukaryotic protein translation	RT		3	7,9	3,2E-3	30,4	7,9E-2	7,9E-2	7,9E-2	9,1E-5
	UP_KW_BIOLOGICAL_PROCESS	Protein biosynthesis	RT		4	10,5	3,3E-3	12,6	7,9E-2	2,7E-2	2,7E-2	2,5E-4
	GOTERM_CC_DIRECT	cortical cytoskeleton	RT		3	7,9	7,0E-4	74,6	9,3E-2	9,7E-3	8,6E-3	8,5E-6
	UP_KW_CELLULAR_COMPONENT	Cytoskeleton	RT		9	23,7	4,6E-3	3,2	9,3E-2	4,9E-2	4,6E-2	1,4E-3
	UP_KW_MOLECULAR_FUNCTION	Actin-binding	RT		5	13,2	4,0E-3	7,3	1,1E-1	3,8E-2	3,6E-2	5,4E-4

Sublist	Category	Term	RT	Genes	Count	%	P-Value	Fold Enrichment	Bonferroni	Benjamini	FDR	Fisher Exact
☐	UP_KW_LIGAND	ATP-binding	RT		9	23,7	1,4E-2	2,4	1,3E-1	6,9E-2	6,9E-2	5,6E-3
☐	GOTERM_MF_DIRECT	actin filament binding	RT		5	13,2	7,7E-4	11,7	1,3E-1	1,4E-2	1,3E-2	6,4E-5
☐	UP_KW_DISEASE	Hereditary hemolytic anemia	RT		3	7,9	7,5E-3	21,2	1,4E-1	1,5E-1	1,5E-1	3,3E-4
☐	KEGG_PATHWAY	Viral carcinogenesis	RT		5	13,2	1,7E-3	9,0	1,5E-1	2,2E-2	1,9E-2	1,8E-4
☐	GOTERM_MF_DIRECT	ubiquitin binding	RT		4	10,5	9,3E-4	20,3	1,6E-1	1,6E-2	1,4E-2	4,4E-5
☐	KEGG_PATHWAY	Lipid and atherosclerosis	RT		5	13,2	2,0E-3	8,6	1,7E-1	2,4E-2	2,1E-2	2,3E-4
☐	UP_KW_MOLECULAR_FUNCTION	Initiation factor	RT		3	7,9	7,0E-3	22,9	1,8E-1	4,9E-2	4,7E-2	2,9E-4
☐	GOTERM_CC_DIRECT	ficolin-1-rich granule lumen	RT		4	10,5	1,5E-3	17,4	1,8E-1	1,8E-2	1,6E-2	8,1E-5
☐	GOTERM_MF_DIRECT	MHC class II protein complex binding	RT		3	7,9	1,2E-3	56,3	2,0E-1	1,8E-2	1,6E-2	2,0E-5
☐	GOTERM_MF_DIRECT	structural constituent of cytoskeleton	RT		4	10,5	1,3E-3	17,9	2,2E-1	1,8E-2	1,6E-2	7,1E-5
☐	GOTERM_MF_DIRECT	ATP hydrolysis activity	RT		6	15,8	1,3E-3	7,0	2,2E-1	1,8E-2	1,6E-2	1,9E-4
☐	GOTERM_MF_DIRECT	ATP binding	RT		10	26,3	2,1E-3	3,3	3,3E-1	2,6E-2	2,3E-2	6,3E-4
☐	GOTERM_MF_DIRECT	phosphoprotein binding	RT		3	7,9	2,3E-3	41,1	3,4E-1	2,6E-2	2,4E-2	5,3E-5
☐	INTERPRO	UBA	RT		3	7,9	2,9E-3	36,5	3,7E-1	7,8E-2	7,5E-2	7,6E-5
☐	GOTERM_MF_DIRECT	transmembrane transporter binding	RT		4	10,5	2,6E-3	14,1	3,9E-1	2,9E-2	2,5E-2	1,8E-4
☐	KEGG_PATHWAY	Fluid shear stress and atherosclerosis	RT		4	10,5	5,4E-3	10,5	4,0E-1	5,6E-2	4,9E-2	5,0E-4
☐	GOTERM_CC_DIRECT	axon	RT		5	13,2	3,6E-3	7,6	4,0E-1	4,3E-2	3,7E-2	4,6E-4
☐	UP_KW_PTM	Isopeptide bond	RT		10	26,3	3,5E-2	2,1	4,2E-1	1,9E-1	1,6E-1	1,6E-2
☐	GOTERM_CC_DIRECT	cortical actin cytoskeleton	RT		3	7,9	4,8E-3	28,3	4,9E-1	4,9E-2	4,3E-2	1,6E-4
☐	GOTERM_MF_DIRECT	hydrolase activity	RT		5	13,2	3,7E-3	7,6	4,9E-1	3,7E-2	3,3E-2	4,7E-4
☐	GOTERM_CC_DIRECT	brush border	RT		3	7,9	4,9E-3	27,8	5,0E-1	4,9E-2	4,3E-2	1,7E-4
☐	GOTERM_MF_DIRECT	protein phosphatase inhibitor activity	RT		3	7,9	3,8E-3	31,7	5,1E-1	3,7E-2	3,3E-2	1,2E-4
☐	GOTERM_MF_DIRECT	actin binding	RT		5	13,2	4,4E-3	7,3	5,5E-1	4,0E-2	3,6E-2	5,8E-4
☐	KEGG_PATHWAY	Tight junction	RT		4	10,5	9,1E-3	8,7	5,7E-1	8,4E-2	7,3E-2	1,0E-3
☐	INTERPRO	UBA-like_sf	RT		3	7,9	5,3E-3	26,9	5,7E-1	1,1E-1	1,0E-1	1,9E-4
☐	UP_KW_PTM	Ubl conjugation	RT		12	31,6	6,1E-2	1,7	6,1E-1	2,5E-1	2,1E-1	3,4E-2
☐	GOTERM_CC_DIRECT	protein-containing complex	RT		6	15,8	7,2E-3	4,8	6,4E-1	6,7E-2	5,9E-2	1,5E-3
☐	GOTERM_MF_DIRECT	translation initiation factor activity	RT		3	7,9	5,7E-3	25,8	6,5E-1	4,8E-2	4,3E-2	2,1E-4
☐	UP_SEQ_FEATURE	CROSSLNK:Glycyl lysine isopeptide (Lys-Gly) (interchain with G-Cter in SUMO2); alternate	RT		5	13,2	3,4E-3	7,8	6,9E-1	2,4E-1	2,4E-1	4,3E-4
☐	KEGG_PATHWAY	Motor proteins	RT		4	10,5	1,4E-2	7,5	7,2E-1	1,1E-1	9,9E-2	1,7E-3
☐	GOTERM_BP_DIRECT	regulation of protein stability	RT		4	10,5	3,1E-3	13,2	7,6E-1	2,2E-1	2,2E-1	2,3E-4
☐	UP_SEQ_FEATURE	DOMAIN:UBA	RT		3	7,9	4,2E-3	30,2	7,6E-1	2,4E-1	2,4E-1	1,3E-4
☐	GOTERM_BP_DIRECT	protein localization	RT		4	10,5	3,2E-3	13,1	7,8E-1	2,2E-1	2,2E-1	2,4E-4
☐	GOTERM_BP_DIRECT	sarcomere organization	RT		3	7,9	3,3E-3	34,3	7,8E-1	2,2E-1	2,2E-1	9,1E-5
☐	GOTERM_MF_DIRECT	protein domain specific binding	RT		4	10,5	8,3E-3	9,3	7,8E-1	6,6E-2	5,9E-2	8,6E-4
☐	KEGG_PATHWAY	Antigen processing and presentation	RT		3	7,9	1,8E-2	13,7	8,2E-1	1,4E-1	1,2E-1	1,3E-3
☐	GOTERM_BP_DIRECT	platelet aggregation	RT		3	7,9	3,7E-3	32,2	8,2E-1	2,2E-1	2,2E-1	1,1E-4
☐	GOTERM_BP_DIRECT	protein folding	RT		4	10,5	4,2E-3	12,0	8,5E-1	2,2E-1	2,2E-1	3,4E-4
☐	GOTERM_CC_DIRECT	actin filament	RT		3	7,9	1,4E-2	16,4	8,5E-1	1,1E-1	9,5E-2	8,1E-4
☐	KEGG_PATHWAY	Cytoskeleton in muscle cells	RT		4	10,5	2,1E-2	6,4	8,6E-1	1,5E-1	1,3E-1	3,1E-3
☐	GOTERM_CC_DIRECT	Schaffer collateral - CA1 synapse	RT		3	7,9	1,4E-2	16,3	8,6E-1	1,1E-1	9,5E-2	8,3E-4
☐	GOTERM_BP_DIRECT	protein destabilization	RT		3	7,9	4,5E-3	29,2	8,7E-1	2,2E-1	2,2E-1	1,5E-4
☐	GOTERM_BP_DIRECT	translational initiation	RT		3	7,9	4,5E-3	29,2	8,7E-1	2,2E-1	2,2E-1	1,5E-4
☐	GOTERM_BP_DIRECT	cellular response to heat	RT		3	7,9	4,8E-3	28,2	8,9E-1	2,2E-1	2,2E-1	1,6E-4
☐	GOTERM_MF_DIRECT	protein phosphatase binding	RT		3	7,9	1,2E-2	17,3	9,0E-1	9,5E-2	8,5E-2	6,9E-4
☐	KEGG_PATHWAY	Salmonella infection	RT		4	10,5	2,6E-2	5,9	9,1E-1	1,6E-1	1,4E-1	4,2E-3
☐	KEGG_PATHWAY	Hypertrophic cardiomyopathy	RT		3	7,9	2,6E-2	11,2	9,2E-1	1,6E-1	1,4E-1	2,3E-3
☐	UP_KW_MOLECULAR_FUNCTION	RNA-binding	RT		5	13,2	8,9E-2	2,8	9,3E-1	4,9E-1	4,7E-1	3,0E-2
☐	GOTERM_CC_DIRECT	secretory granule lumen	RT		3	7,9	1,9E-2	13,9	9,3E-1	1,3E-1	1,1E-1	1,3E-3
☐	KEGG_PATHWAY	Dilated cardiomyopathy	RT		3	7,9	3,0E-2	10,6	9,4E-1	1,7E-1	1,5E-1	2,7E-3
☐	GOTERM_CC_DIRECT	cytoskeleton	RT		5	13,2	2,1E-2	4,6	9,5E-1	1,4E-1	1,2E-1	4,3E-3
☐	GOTERM_BP_DIRECT	response to unfolded protein	RT		3	7,9	6,4E-3	24,3	9,5E-1	2,4E-1	2,4E-1	2,5E-4
☐	KEGG_PATHWAY	Apoptosis	RT		3	7,9	4,7E-2	8,2	9,9E-1	2,5E-1	2,2E-1	5,6E-3
☐	GOTERM_MF_DIRECT	histone deacetylase binding	RT		3	7,9	2,5E-2	11,9	9,9E-1	1,7E-1	1,5E-1	2,0E-3
☐	KEGG_PATHWAY	Estrogen signaling pathway	RT		3	7,9	4,9E-2	8,0	9,9E-1	2,5E-1	2,2E-1	5,9E-3
☐	GOTERM_MF_DIRECT	unfolded protein binding	RT		3	7,9	2,5E-2	11,8	9,9E-1	1,7E-1	1,5E-1	2,1E-3
☐	GOTERM_CC_DIRECT	vesicle	RT		3	7,9	3,4E-2	10,0	9,9E-1	2,0E-1	1,8E-1	3,3E-3
☐	GOTERM_CC_DIRECT	membrane	RT		16	42,1	3,8E-2	1,6	1,0E0	2,1E-1	1,8E-1	2,3E-2

Sublist	Category	Term	RT	Genes	Count	%	P-Value	Fold Enrichment	Bonferroni	Benjamini	FDR	Fisher Exact
	INTERPRO	Papain-like_cys_pep_sf	RT		3	7,9	3,9E-2	9,3	1,0E0	3,2E-1	3,1E-1	4,1E-3
	GOTERM_CC_DIRECT	lamellipodium	RT		3	7,9	4,5E-2	8,6	1,0E0	2,3E-1	2,0E-1	5,1E-3
	GOTERM_CC_DIRECT	cell-cell junction	RT		3	7,9	4,6E-2	8,6	1,0E0	2,3E-1	2,0E-1	5,2E-3
	GOTERM_BP_DIRECT	response to endoplasmic reticulum stress	RT		3	7,9	1,4E-2	16,1	1,0E0	3,6E-1	3,6E-1	8,5E-4
	GOTERM_CC_DIRECT	midbody	RT		3	7,9	4,7E-2	8,4	1,0E0	2,3E-1	2,0E-1	5,4E-3
	KEGG_PATHWAY	Influenza A	RT		3	7,9	7,2E-2	6,4	1,0E0	3,5E-1	3,1E-1	1,1E-2
	GOTERM_CC_DIRECT	presynapse	RT		3	7,9	5,1E-2	8,0	1,0E0	2,3E-1	2,0E-1	6,2E-3
	GOTERM_CC_DIRECT	synapse	RT		4	10,5	6,7E-2	4,1	1,0E0	2,8E-1	2,4E-1	1,5E-2
	GOTERM_MF_DIRECT	calcium ion binding	RT		5	13,2	5,2E-2	3,4	1,0E0	3,4E-1	3,1E-1	1,4E-2
	GOTERM_CC_DIRECT	mitochondrion	RT		7	18,4	8,1E-2	2,2	1,0E0	3,2E-1	2,8E-1	3,4E-2
	GOTERM_BP_DIRECT	proteolysis	RT		5	13,2	2,6E-2	4,3	1,0E0	5,1E-1	5,1E-1	5,8E-3
	GOTERM_BP_DIRECT	regulation of cell shape	RT		3	7,9	2,6E-2	11,5	1,0E0	5,1E-1	5,1E-1	2,2E-3
	GOTERM_CC_DIRECT	chromosome	RT		3	7,9	8,9E-2	5,8	1,0E0	3,2E-1	2,8E-1	1,5E-2
	GOTERM_BP_DIRECT	actin filament organization	RT		3	7,9	2,9E-2	10,9	1,0E0	5,2E-1	5,2E-1	2,6E-3
	GOTERM_BP_DIRECT	signal transduction	RT		7	18,4	3,1E-2	2,8	1,0E0	5,2E-1	5,2E-1	1,1E-2
	GOTERM_MF_DIRECT	magnesium ion binding	RT		3	7,9	7,7E-2	6,4	1,0E0	4,7E-1	4,2E-1	1,2E-2
	GOTERM_BP_DIRECT	cytoskeleton organization	RT		3	7,9	3,4E-2	10,1	1,0E0	5,2E-1	5,2E-1	3,2E-3
	GOTERM_BP_DIRECT	negative regulation of apoptotic process	RT		4	10,5	6,9E-2	4,1	1,0E0	7,8E-1	7,8E-1	1,6E-2
	GOTERM_BP_DIRECT	regulation of apoptotic process	RT		3	7,9	8,2E-2	6,1	1,0E0	8,4E-1	8,4E-1	1,3E-2

Please cite DAVID within any publication that makes use of any methods inspired by **DAVID**.
